# Near‐Infrared‐Plasmonic Energy Upconversion in a Nonmetallic Heterostructure for Efficient H_2_ Evolution from Ammonia Borane

**DOI:** 10.1002/advs.201800748

**Published:** 2018-07-03

**Authors:** Zhenyi Zhang, Yang Liu, Yurui Fang, Baosheng Cao, Jindou Huang, Kuichao Liu, Bin Dong

**Affiliations:** ^1^ Key Laboratory of New Energy and Rare Earth Resource Utilization of State Ethnic Affairs Commission Key Laboratory of Photosensitive Materials & Devices of Liaoning Province School of Physics and Materials Engineering Dalian Minzu University 18 Liaohe West Road Dalian 116600 P. R. China; ^2^ School of Materials Science and Engineering Dalian University of Technology Dalian 116024 P. R. China; ^3^ Key Laboratory of Materials Modification by Laser Electron and Ion Beams (Ministry of Education) School of Physics Dalian University of Technology Dalian 116024 P. R. China

**Keywords:** H_2_ evolution, lanthanide ions, plasmonic semiconductors, upconversion luminescence

## Abstract

Plasmonic metal nanostructures have been widely used to enhance the upconversion efficiency of the near‐infrared (NIR) photons into the visible region via the localized surface plasmon resonance (LSPR) effect. However, the direct utilization of low‐cost nonmetallic semiconductors to both concentrate and transfer the NIR‐plasmonic energy in the upconversion system remains a significant challenge. Here, a fascinating process of NIR‐plasmonic energy upconversion in Yb^3+^/Er^3+^‐doped NaYF_4_ nanoparticles (NaYF_4_:Yb‐Er NPs)/W_18_O_49_ nanowires (NWs) heterostructures, which can selectively enhance the upconversion luminescence by two orders of magnitude, is demonstrated. Combined with theoretical calculations, it is proposed that the NIR‐excited LSPR of W_18_O_49_ NWs is the primary reason for the enhanced upconversion luminescence of NaYF_4_:Yb‐Er NPs. Meanwhile, this plasmon‐enhanced upconversion luminescence can be partly absorbed by the W_18_O_49_ NWs to re‐excite its higher energy LSPR, thus leading to the selective enhancement of upconversion luminescence for the NaYF_4_:Yb‐Er/W_18_O_49_ heterostructures. More importantly, based on this process of plasmonic energy transfer, an NIR‐driven catalyst of NaYF_4_:Yb‐Er NPs@W_18_O_49_ NWs quasi‐core/shell heterostructure, which exhibits a ≈35‐fold increase in the catalytic H_2_ evolution from ammonia borane (BH_3_NH_3_) is designed and synthesized. This work provides insight on the development of nonmetallic plasmon‐sensitized optical materials that can potentially be applied in photocatalysis, optoelectronic, and photovoltaic devices.

Over the past several decades, the fascinating photophysical phenomena of localized surface plasmon resonance (LSPR) have attracted significant interests in research areas such as electronics, photonics, and catalysis. This is mainly due to their unique capability to concentrate and amplify the incident light intensity near the surface of plasmonic nanostructures.^1^, ^2^, ^3^ As a classic plasmonic “optical antenna,” noble metal nanostructures with tunable LSPR energy are frequently introduced into nanomaterials to promote their performance in light absorption and/or emission via plasmonic energy transfer from noble metal to neighboring optical nanomaterials.^4^, ^5^, ^6^ For example, coupling appropriate nanostructures of plasmonic Au or Ag with upconversion nanomaterials, in particular trivalent lanthanide ions (Ln^3+^)‐doped NaYF_4_ nanoparticles (NPs), can achieve enhanced upconversion luminescence in the visible light region by converting lower frequency incident photons at 980 nm with high effectivity.^7^, ^8^, ^9^ This paradigm has been regarded as a promising tactic to use the low‐energy NIR light, which makes up almost half of the solar energy (≈40%), in the field of solar‐to‐fuels energy conversion.^10^, ^11^ From a plasmonic point of view, the overlapping spectra between LSPR bands of noble metals and the excitation/emission bands of upconversion NPs would result in a highly efficient upconversion of NIR to visible light.^12^, ^13^ Although some specific metallic structures, such as long nanowires (NWs) and nanoforest, can easily provide a very wide band for simultaneously matching the excitation and emission bands of Ln^3+^‐doped NaYF_4_ NPs, manipulating the shapes and sizes of these metallic structures often requires a very complex synthesis process.^14^, ^15^ Furthermore, using noble metals inevitably suffers from high cost and earth rarity. Therefore, exploiting noble‐metal‐free tractable plasmonic nanostructures to sensitize the upconversion luminescence of Ln^3+^‐doped NaYF_4_ NPs is of great significance for boosting the development of NIR‐driven energy generation.

Recently, some researches have reported that the LSPR phenomena can also occur on various nonmetal nanostructures of heavily doped nonstoichiometric semiconductors, such as Cu_2−_ *_x_*S, Sn‐doped In_2_O_3_, WO_3−_ *_x_*, and MoO_3−_ *_x_*.^16^, ^17^, ^18^, ^19^, ^20^ LSPR band and intensity of the semiconductor can be easily controlled via adjusting the stoichiometric ratios, vacancy, or dopant concentrations, as well as phase structures.^21^, ^22^, ^23^ Among the plasmonic semiconductor nanostructures, tungsten oxide NWs, W_18_O_49_, made via a facile solvothermal method, possess intense LSPR band across the visible and NIR regions due to abundant oxygen vacancies on their surface.^24^, ^25^, ^26^ Such a broad plasmonic absorption of W_18_O_49_ NWs covers both excitation and emission spectra of Yb^3+^/Er^3+^‐doped NaYF_4_ (NaYF_4_:Yb‐Er) NPs, which offers an unique opportunity to gain deeper understanding on the influence of the LSPR on the photon absorption and emission processes of the upconversion NPs within the same plasmonic nanostructure. Moreover, plasmonic W_18_O_49_ NWs have demonstrated outstanding catalytic performance for the H_2_ evolution from ammonia borane (BH_3_NH_3_).^27^ Combining plasmonic W_18_O_49_ NWs with NaYF_4_:Yb‐Er NPs to form a hierarchical heterostructure would also promote the catalytic activity for H_2_ evolution due to the LSPR‐induced interaction between both nanomaterials. This may reveal a new way to explore the NIR‐active catalyst for potential applications for the development of sustainable energy sources.

For the first time, we demonstrate the nonmetallic plasmon‐induced selective enhancement of upconversion luminescence in a layer‐structured film consisting of NaYF_4_:Yb‐Er NPs as the upconversion layer and W_18_O_49_ NWs as the plasmonic layer. Compared with the individual NaYF_4_:Yb‐Er film, the NaYF_4_:Yb‐Er/W_18_O_49_ film exhibited two orders upconversion enhancement on the green emission originating from the ^2^H_11/2_–^4^I_15/2_ transition of Er^3+^ ions, but a decreased effect from red emission, which was ascribed to Er^3+^ ions with the ^4^F_9/2_–^4^I_15/2_ transition. Combined with the theoretical calculations, we propose that this selective enhancement of upconversion luminescence is caused via LSPR‐induced NIR‐plasmonic energy upconversion. In this process, W_18_O_49_ NWs convert the NIR energy of incident photons into LSPR oscillation energy that subsequently transfers to the adjacent NaYF_4_:Yb‐Er NPs, thus substantially enhancing the upconversion luminescence intensity based on the plasmon‐enhanced localized electric fields and the photothermally improved electron population. The enhanced upconversion luminescence of NaYF_4_:Yb‐Er NPs would then re‐excite the LSPR oscillations of W_18_O_49_ NWs in the visible region, resulting in the selective absorption of upconversion luminescence depending on the LSPR band of W_18_O_49_ NWs (**Scheme** **1**). Due to this unique plasmonic energy upconversion phenomenon, an enhanced catalytic activity for H_2_ evolution from BH_3_NH_3_ was achieved over the NaYF_4_:Yb‐Er NPs@W_18_O_49_ NWs quasi‐core/shell structure synthesized via facile solvothermal method.

**Scheme 1:**
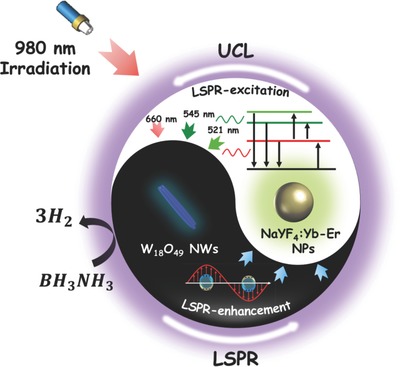
Schematic diagram of plasmonic energy upconversion in the NaYF_4_:Yb‐Er NPs/W_18_O_49_ NWs system upon irradiation at 980 nm.

To clarify the influence of W_18_O_49_ LSPR on the upconversion behavior of NaYF_4_:Yb‐Er NPs, a binary film with layer‐on‐layer heterostructure was designed and constructed hierarchically onto F‐doped SnO_2_ (FTO) glass. The FTO glass is chosen as the film substrate, because it contains abundant active sites for benefiting the uniform growth of plasmonic W_18_O_49_ NWs on its surface. **Figure** **1**a shows the schematic illustration of the two‐step assembly route and the profile of the NaYF_4_:Yb‐Er/W_18_O_49_ film. During the first step, W_18_O_49_ NWs were grown onto the FTO glass to achieve the plasmonic layer via a solvothermal process; for the second step, the NaYF_4_:Yb‐Er NPs were self‐assembled on the top surface of the W_18_O_49_ NWs layer through a solvent evaporation process, therefore forming a thin upconversion‐luminescence layer above the plasmonic layer. Meanwhile, two control samples were constructed on the FTO glass: one is the NaYF_4_:Yb‐Er film, which was fabricated by direct self‐assembly of NaYF_4_:Yb‐Er NPs onto the FTO glass; the other is the NaYF_4_:Yb‐Er/N‐W_18_O_49_ film, where the N‐W_18_O_49_ NWs, denoting non‐plasmonic W_18_O_49_ NWs, were obtained through the H_2_O_2_ treatment of plasmonic W_18_O_49_ NWs to passivate their surface oxygen vacancies.

**Figure 1:**
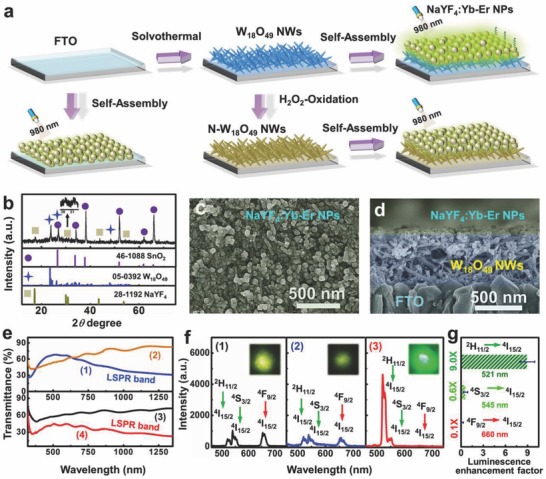
a) Fabrication processes of the NaYF_4_:Yb‐Er, NaYF_4_:Yb‐Er/W_18_O_49_, and NaYF_4_:Yb‐Er/N‐W_18_O_49_ films on FTO glass. b) XRD pattern of the as‐fabricated NaYF_4_:Yb‐Er/W_18_O_49_ film. SEM images of c) the top view and d) the side view of the NaYF_4_:Yb‐Er/W_18_O_49_ film. e) Transmittance spectra of 1) W_18_O_49_ NWs, 2) N‐W_18_O_49_ NWs, 3) NaYF_4_:Yb‐Er/N‐W_18_O_49_, and 4) NaYF_4_:Yb‐Er/W_18_O_49_ films. f) Upconversion emission spectra of 1) NaYF_4_:Yb‐Er, 2) NaYF_4_:Yb‐Er/N‐W_18_O_49_, and 3) NaYF_4_:Yb‐Er/W_18_O_49_ films. The insets provide the corresponding micro‐area optical images achieved under irradiation at 980 nm. g) Histogram of enhancement factors calculated via the luminescence intensity ratios of NaYF_4_:Yb‐Er/W_18_O_49_ film to the NaYF_4_:Yb‐Er film at the different emission wavelengths.

The X‐ray diffraction (XRD) pattern of the NaYF_4_:Yb‐Er/W_18_O_49_ film shows three sets of characteristic peaks, belonging to hexagonal β‐NaYF_4_, monoclinic W_18_O_49_, and tetragonal SnO_2_, respectively (Figure 1b). Scanning electron microscopic (SEM) images display both top surface (Figure 1c) and cross section (Figure 1d) of the above film. It can be seen that NaYF_4_:Yb‐Er NPs with a mean size of ≈40 nm are interconnected and cover the top surface of the composite film, thus forming a thin upconversion‐luminescence layer. Furthermore, W_18_O_49_ NWs with diameters of 20–40 nm and lengths of 0.5–1.0 µm aligned in random orientations on the FTO glass, where they interweaved to compose a plasmonic layer (of ≈500 nm thickness) below the upconversion‐luminescence layer. The randomly tilted growth of W_18_O_49_ NWs on the film enabled the majority of self‐assembled NaYF_4_:Yb‐Er NPs to directly contact the side surface of plasmonic W_18_O_49_ NWs. The control sample of NaYF_4_:Yb‐Er/N‐W_18_O_49_ film was identical to the NaYF_4_:Yb‐Er/W_18_O_49_ film in structural features and component morphology. Furthermore, the NaYF_4_:Yb‐Er film possessed similar thickness as the upconversion luminescence layer in the NaYF_4_:Yb‐Er/W_18_O_49_ film (Figure S1, Supporting Information). Figure 1e presents the comparison transmittance spectra of the plasmonic W_18_O_49_‐based film and the N‐W_18_O_49_‐based film on the FTO glass. The plasmonic W_18_O_49_ NWs grown on the FTO glass exhibited a low transmittance in the visible–NIR region. Meanwhile, the transmittance is dependent on the incident wavelength. These observations are in accordance with the intrinsic optical property of W_18_O_49_ reported in the literatures,^25^, ^26^, ^27^ confirming that the low transmittance of W_18_O_49_‐based film is originated from intense LSPR absorption of the W_18_O_49_ NWs. The LSPR of W_18_O_49_ NWs could remain at least three months at room temperature and atmosphere pressure (Figure S1, Supporting Information), even though this property derives from the oxygen vacancies. After treatment with H_2_O_2_ to form N‐W_18_O_49_ NWs, the LSPR absorption disappeared. Importantly, when loading a thin layer of NaYF_4_:Yb‐Er NPs on the layer of plasmonic W_18_O_49_ NWs, a distinct LSPR band can still be observed on the spectra of the NaYF_4_:Yb‐Er/W_18_O_49_ film. However, the NaYF_4_:Yb‐Er/N‐W_18_O_49_ film did not show the plasmonic absorption band in the visible–NIR region even though it shared the same component morphology with the NaYF_4_:Yb‐Er/W_18_O_49_ film.

Upconversion‐luminescence properties of the as‐fabricated films were tested via an inverted microscope coupled with a spectrometer and a 980 nm laser diode (Scheme S2, Supporting Information). It is clear that two green emission peaks and one red emission peak appearing on the spectrum of the NaYF_4_:Yb‐Er film (Figure 1f) are attributed to ^2^H_11/2_ → ^4^I_15/2_, ^4^S_3/2_ → ^4^I_15/2_, and ^4^F_9/2_ → ^4^I_15/2_ transitions of Er^3+^ ions, respectively.^7^, ^8^, ^9^ Also note that the intensity ratio of green‐to‐red emission (*I*_green_/*I*_red_) is only ≈1.4, resulting in an overall green‐yellow color output (see inset of Figure 1f). In the case of the NaYF_4_:Yb‐Er/N‐W_18_O_49_ film without plasmonic absorption property, the ratio value of *I*_green_/*I*_red_ is ≈1.7, with the emission peak profiles almost identical to the corresponding parameters within the NaYF_4_:Yb‐Er only film. This implies that the upconversion behavior of NaYF_4_:Yb‐Er NPs is not influenced by the N‐W_18_O_49_ NWs layer in the composite film. To our surprise, when these N‐W_18_O_49_ NWs are replaced with the plasmonic W_18_O_49_ NWs in the composite film, the NaYF_4_:Yb‐Er/W_18_O_49_ film exhibits a remarkable enhancement in the intensity for green emission; in particular, the emission peak at 521 nm was nearly one order of magnitude (9.0×) higher than the corresponding emission intensity of the NaYF_4_:Yb‐Er film (Figure 1g). However, the red emission at 660 nm decreased to 10% of the NaYF_4_:Yb‐Er film. As a result, the *I*_green_/*I*_red_ value of NaYF_4_:Yb‐Er NPs increased to ≈75 after introducing the plasmonic W_18_O_49_ NWs layer into the composite film. Thus, a bright green emission became noticeable on the micro‐area photograph of the NaYF_4_:Yb‐Er/W_18_O_49_ film in response to excitation with 980 nm (see inset of Figure 1f). It is apparent that a selective enhancement of green upconversion emission was found on the NaYF_4_:Yb‐Er/W_18_O_49_ film.

When the NaYF_4_:Yb‐Er NPs were deposited on the plasmonic W_18_O_49_ NW surface in the composite film, a strong interaction between LSPR and upconversion optical field occurred at their interface since the LSPR band of W_18_O_49_ NWs overlapped with both the excitation and emission electric fields of NaYF_4_:Yb‐Er NPs (see Figure 1e). Upon 980 nm excitation, the W_18_O_49_ NWs serve as the “plasmonic antenna” to locally concentrate the NIR energy near the NWs, and then resonantly transfer this energy to adjoining NaYF_4_:Yb‐Er NPs (**Figure** **2**a).^28^, ^29^ Therefore, the NaYF_4_:Yb‐Er NPs near the NaYF_4_:Yb‐Er/W_18_O_49_ interface would experience a far more intense excitation electric field based on the surface enhancement effect, which could promote electron population on the excited‐state energy levels of Er^3+^ ions, thus resulting in an enhanced upconversion luminescence.^4^, ^7^, ^30^ Meanwhile, the LSPR‐enhanced localized electric field can interact with the emission electric field of NaYF_4_:Yb‐Er NPs to boost the radiative decay rate of upconversion process.^4^, ^7^ However, the direct contact of W_18_O_49_ NWs and NaYF_4_:Yb‐Er NPs quenches the upconversion emission to a certain degree due to the nonradiative energy transfer from the NaYF_4_:Yb‐Er NPs (donor) and the adherent W_18_O_49_ NWs (acceptor).^31^, ^32^, ^33^, ^34^ Moreover, the upconversion luminescence of NaYF_4_:Yb‐Er NPs can also be absorbed by the adjacent W_18_O_49_ NWs, which selectively weakens the luminescence intensity depending on the absorption band of W_18_O_49_ NWs. Furthermore, the LSPR‐induced photothermal effect of W_18_O_49_ NWs can also raise the local temperature of NaYF_4_:Yb‐Er NPs to affect their upconversion process. Based on the above considerations, we proposed that the photophysical mechanism (Figure 2a) for this selective enhancement of upconversion luminescence is attributed to the following three aspects:

1) Plasmon‐mediated competition between the radiative and nonradiative processes: We employed the finite element method to simulate the localized electric field intensity and distribution near the NaYF_4_:Yb‐Er NPs by plasmon‐excitation of W_18_O_49_ NWs. The transmission electron microscopy (TEM) image suggests that the W_18_O_49_ NWs have a bundle‐like nanostructure, consisting of several ultrathin secondary NWs with diameters of ≈10 nm (Figure S2a, Supporting Information). The TEM image of the NaYF_4_:Yb‐Er NPs confirmed their hexagonal nanostructure with a mean size of ≈40 nm (Figure S2b, Supporting Information). To simplify the calculation, a binary heterostructure is proposed in the simulation model, in which the single NaYF_4_ NP is selectively loaded on the four representative positions of the bundle‐like W_18_O_49_ NWs surface. As illustrated in the inset of Figure 2b, P1 and P2 represent the positions at the quarter and the half of W_18_O_49_ NWs, respectively, which are symmetric to the corresponding sites of P3 and P4. We first simulated the localized electric field distributions of the above four types of NaYF_4_/W_18_O_49_ heterostructures under excitation of 980 nm. When the NaYF_4_ NP was tangential to one of the NWs at P1 or P2 on the W_18_O_49_ bundle surface, the maximum electric field intensity enhancement (|*E*|^2^/|*E*_0_|^2^) could reach ≈29 at the plasmonic “hot spots,” i.e., in the vicinity of the NaYF_4_/W_18_O_49_ interface (Figure 2b; Figure S2c, Supporting Information). Furthermore, if the NaYF_4_ NP was deposited on either P3 or P4 to tangent simultaneously two of the NWs in the W_18_O_49_ bundle, it can obtain a ≈14‐fold increase of the excitation electric field intensity at the NaYF_4_/W_18_O_49_ interface (Figure S2c, Supporting Information). Interestingly, the distal edges of NaYF_4_ NPs, opposite from the plasmonic “hot spots,” also showed about a two‐ to four‐fold enhancement of the localized electric field. Also note that the LSPR‐enhanced excitation field concentrates mainly on the NaYF_4_/W_18_O_49_ interface with a depth below ≈10 nm towards the NaYF_4_. Second, interactions of W_18_O_49_ LSPR with the emission electric field of NaYF_4_ NP were assessed through simulating the electric field distributions with emission wavelengths at 521, 545, and 660 nm, respectively (Figures S3–S5, Supporting Information). The maximum enhancement on the emission electric field intensities of the heterostructures also occurs near the NaYF_4_/W_18_O_49_ interface. Moreover, the enhancement factors at different emission wavelengths fluctuate in a range from 3.4 to 8.0, depending on the contact sites of NaYF_4_ NPs on the W_18_O_49_ NWs surface. It should be also noted that the emission electric field enhancement tendency and distributions close to the NaYF_4_ NPs almost overlapped with the corresponding features of the excitation electric field enhancement induced by the W_18_O_49_ LSPR. As such, the overall enhancement of optical field at plasmonic “hot spots” of the NaYF_4_/W_18_O_49_ heterostructures ranged from 70 to 220 times (Figure S6, Supporting Information).

**Figure 2:**
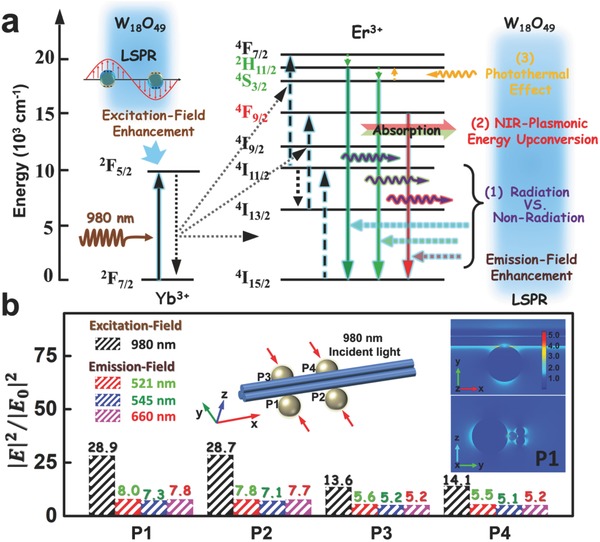
a) Schematic diagram of the interaction between the LSPR effect of W_18_O_49_ NWs and the energy‐transfer upconversion process of NaYF_4_:Yb‐Er NPs. b) Simulated enhancements of excitation (980 nm) and emission electric‐field intensities (521, 545, and 660 nm) at the plasmonic “hot spots” as a function of the contact positions between the NaYF_4_ NPs and W_18_O_49_ NWs. Insets show the simulation model of the NaYF_4_/W_18_O_49_ heterostructure with the NaYF_4_ NPs loaded on the representative positions of the W_18_O_49_ NWs surface; and the simulation of excitation electric field distribution at position P1 with input irradiation along the *y*‐axis.

However, in our case, the NaYF_4_ NPs are directly in contact with the plasmonic W_18_O_49_ NWs and the nonradiative energy transfer to W_18_O_49_ NWs provides an efficient decay channel to quench the upconversion luminescence.^31^, ^32^, ^33^, ^34^ The time‐resolved luminescence spectroscopy indicated that the lifetimes of ^2^I_11/2_ → ^4^I_15/2_ (521 nm), ^4^S_3/2_ → ^4^I_15/2_ (545 nm), and ^4^F_9/2_–^4^I_15/2_ (660 nm) decays for the NaYF_4_:Yb‐Er/W_18_O_49_ film were shorter than the corresponding lifetimes obtained from the individual NaYF_4_:Yb‐Er film (Figure S7, Supporting Information). Combining with the results of selective luminescence enhancement, we confirmed the existence of plasmon‐mediated competition between the radiative and nonradiative processes in the NaYF_4_:Yb‐Er/W_18_O_49_ film after 980 nm excitation.^4^, ^7^ The radiative rate enhancement is originated from the plasmon‐enhanced emission field, while the nonradiative process in the NaYF_4_:Yb‐Er/W_18_O_49_ film is mainly attributed to the mentioned energy transfer. This nonradiative energy transfer is strongly dependent on the distance between luminescent centers and plasmonic nanostructures.^33^, ^34^ For NaYF_4_:Yb‐Er NPs, the luminescent centers of Er^3+^ ions are uniformly dispersed in the insulating NaYF_4_ host.^30^ Thus, the emission quenching process should occur only on the Er^3+^ ions located nearby the NaYF_4_:Yb‐Er/W_18_O_49_ interface. A large number of luminescent centers in the NaYF_4_:Yb‐Er NPs could enjoy the LSPR‐enhanced excitation and emission fields with the weakened quenching effect, therefore leading to the enhancement of upconversion luminescence on the NaYF_4_:Yb‐Er/W_18_O_49_ film. To further confirm the existence of nonradiative energy transfer in the NaYF_4_:Yb‐Er/W_18_O_49_ film, we introduced the insulating spacer with different thicknesses to separate the NaYF_4_:Yb‐Er and W_18_O_49_ components, for the purpose of adjusting the energy transfer process (Figure S8, Supporting Information). Interestingly, when the thickness of the insulating spacer is proper, the upconversion luminescence of NaYF_4_:Yb‐Er NPs in the composite film could be further enhanced due to the suppressed nonradiative energy transfer (Figure S8, Supporting Information). Meanwhile, both the green and red emission enhancements were observed on the NaYF_4_:Yb‐Er/W_18_O_49_ film.

To gain a better understanding on the above plasmon‐mediated competition process, we established the mathematical model based on a set of rate equations (Figure S9 and Equations (S1)–(S20), Supporting Information). The results demonstrated that the LSPR‐enhanced excitation and emission fields were responsible for boosting the upconversion luminescence of NaYF_4_:Yb‐Er NPs in the composite film. However, the transfer process of nonradiative energy competed against the LSPR‐enhanced emission field to quench the upconversion luminescence. Importantly, we concluded that both the emission field enhancement and the nonradiative energy transfer contributed to the enlargement of intensity ratio between the green and red emissions, leading to a relative strong green emission as compared to the light emission from the NaYF_4_:Yb‐Er/W_18_O_49_ film.

2) NIR‐plasmonic energy upconversion: The LSPR‐enhanced upconversion luminescence of NaYF_4_:Yb‐Er NPs can be partially absorbed by the adjacent W_18_O_49_ NWs due to their spectral overlap, which induces re‐excitation of the LSPR effect of W_18_O_49_ NWs in the visible region, achieving an NIR‐plasmonic energy upconversion process. According to the absorption spectrum of W_18_O_49_ NWs, the red emission from the NaYF4:Yb‐Er NPs should be more easily absorbed by the W_18_O_49_ NWs as compared to the emitted green light, which leads to a relatively strong green emission of NaYF_4_:Yb‐Er NPs on the W_18_O_49_ NWs film. When the W_18_O_49_ NWs layer did not possess the LSPR band in the visible region, both the green and red emission enhancements could be observed on the NaYF_4_:Yb‐Er/W_18_O_49_ film (Figure S9, Supporting Information). Thus, the NIR‐plasmonic energy upconversion is one of the reasons for the selective absorption of upconversion luminescence.
3) Photothermal effect: After LSPR excitation by 980 nm, the W_18_O_49_ NWs can create a super‐high temperature surrounding their surfaces (similar to the noble metal nanostructures),^34^, ^35^ which would influence the upconversion luminescence of the neighboring NaYF_4_:Yb‐Er NPs in the composite film. It is well known that in the case of NaYF_4_:Yb‐Er, the energy separation (≈840 cm^−1^) between ^2^H_11/2_ and ^4^S_3/2_ levels can allow the thermally excited population from the ^4^S_3/2_ to ^2^H_11/2_ level, and a quasi‐thermal equilibrium forms between these two levels, resulting in the variation in the transitions of ^2^I_11/2_ → ^4^I_15/2_ (521 nm) and ^4^S_3/2_ → ^4^I_15/2_ (545 nm) at an increased temperature (Equation (S21), Supporting Information).^36^, ^37^, ^38^ As observed in Figure 1f, the NaYF_4_:Yb‐Er/W_18_O_49_ film in our case shows a very large ratio of the green upconversion emissions from the ^2^I_11/2_ → ^4^I_15/2_ (521 nm) and ^4^S_3/2_ → ^4^I_15/2_ (545 nm) transitions, which suggests the high temperature located at the near‐surface of NaYF_4_:Yb‐Er NPs due to the photothermal effect of W_18_O_49_ NWs.

Overall, this selective enhancement of upconversion luminescence over the NaYF_4_:Yb‐Er/W_18_O_49_ film was essentially caused by the plasmon‐induced energy transfer between the plasmonic W_18_O_49_ NWs and NaYF_4_:Yb‐Er NPs (Figure 2a).

It is worth noting that the LSPR‐enhanced near‐filed effect mainly occurred at the very near region of the NaYF_4_/W_18_O_49_ interface, which accounts for a small part of the luminescent centers in the NaYF_4_ NP. Certainly, if the size of NaYF_4_ NP reduces to allow more luminescent centers to locate in the plasmonic field, a more effective enhancement of upconversion luminescence is expected on the NaYF_4_/W_18_O_49_ film. As we reduced the grain sizes of NaYF_4_:Yb‐Er NPs in the NaYF_4_/W_18_O_49_ film, the enhancement factor of upconversion luminescence increased dramatically (**Figure** **3**a; Figure S11, Supporting Information). Grain size reduction ensured that the major part of luminescent centers in the NaYF_4_ NP was positioned within the effective interaction distance to enjoy the LSPR‐enhanced field, leading to improved enhancement effect on the upconversion luminescence. Further investigation found that the enhancement factors of ^2^I_11/2_ → ^4^I_15/2_ transitions for all the samples were higher than those of ^4^S_3/2_ → ^4^I_15/2_ transitions. It reveals that the selective enhancement of upconversion luminescence of NaYF_4_ NPs is due to the LSPR of W_18_O_49_ NWs in their composite film. Meanwhile, the optimal enhancement factor of 112× was achieved on the ^2^I_11/2_ → ^4^I_15/2_ transition in 10 nm NaYF_4_:Yb‐Er NPs (Figure 3a). Although 10 nm NaYF_4_:Yb‐Er NPs exhibited optimal enhancement effect in our study, 40 nm NaYF_4_:Yb‐Er NPs showed the largest luminescent intensity after plasmonic sensitization via W_18_O_49_ NWs (Figure 3b).

**Figure 3:**
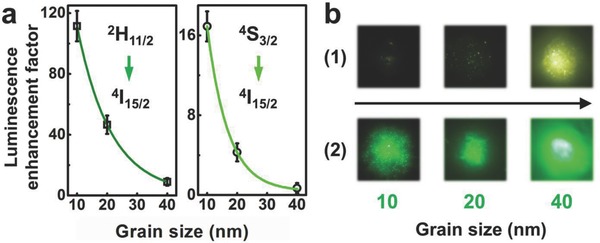
a) Luminescence enhancement factor of ^2^H_11/2_ and ^4^S_3/2_ to ^4^I_15/2_ transitions as a function of the grain sizes of NaYF_4_:Yb‐Er NPs; b) micro‐area optical images of the as‐fabricated films loaded with different sizes of upconversion NPs under irradiation at 980 nm: 1) NaYF_4_:Yb‐Er films; 2) NaYF_4_:Yb‐Er/W_18_O_49_ films.

Plasmonic W_18_O_49_ NWs are an effective catalyst for boosting the H_2_ release from BH_3_NH_3_ via catalytic hydrolysis.^21^, ^27^, ^39^ Coupling plasmonic W_18_O_49_ NWs with Ln^3+^‐doped NaYF_4_ NPs to construct a hierarchical heterostructure would result in a highly efficient H_2_ evolution due to the NIR‐plasmonic energy upconversion process. Considering that the catalytic active sites locate on the W_18_O_49_ NWs surface, a quasi‐core/shell heterostructured catalyst was designed and synthesized via solvothermal growth of W_18_O_49_ NWs onto hydrophilic NaYF_4_:Yb‐Er NPs with a grain size of ≈40 nm (**Figure** **4**a). The as‐synthesized NaYF_4_:Yb‐Er@W_18_O_49_ nanocomposite possesses a spherical structure that exposes numerous NWs on the surface, thus forming a “sea urchin‐like” catalyst (Figure 4b). The diameters of the NaYF_4_:Yb‐Er@W_18_O_49_ heterostructures were around 1 µm. The TEM image shows that the NWs on the heterostructure surface are 400–600 nm in length and 10–30 nm in diameter. The TEM elemental mapping of an individual NaYF_4_:Yb‐Er@W_18_O_49_ heterostructure presents that the Na, Y, and F elements are mostly distributed in the center of the heterostructure, while the W and O elements are present throughout the whole profile, indicating the formation of the quasi‐core/shell heterostructure (Figure 4c). The characterization results of the selected area electron diffraction (SAED) and XRD patterns unambiguously confirm that the as‐synthesized heterostructures consisted of the cubic α‐NaYF_4_ and the monoclinic W_18_O_49_ (Figure 4d,e). Notably, the NaYF_4_:Yb‐Er@W_18_O_49_ heterostructure still displayed an intense LSPR absorption band that overlaps with both the excitation and emission spectra of the NaYF_4_:Yb‐Er NPs (Figure 4f). Taking this LSPR feature, the NaYF_4_:Yb‐Er@W_18_O_49_ heterostructures also exhibited selectively enhanced upconversion emission, as deduced from the normalized emission spectra (Figure 4g). This suggests that the as‐proposed NIR‐plasmonic energy upconversion process occurs in the NaYF_4_:Yb‐Er@W_18_O_49_ heterostructure, which could offer a new and efficient way to enhance the catalytic H_2_ evolution from BH_3_NH_3_.

**Figure 4:**
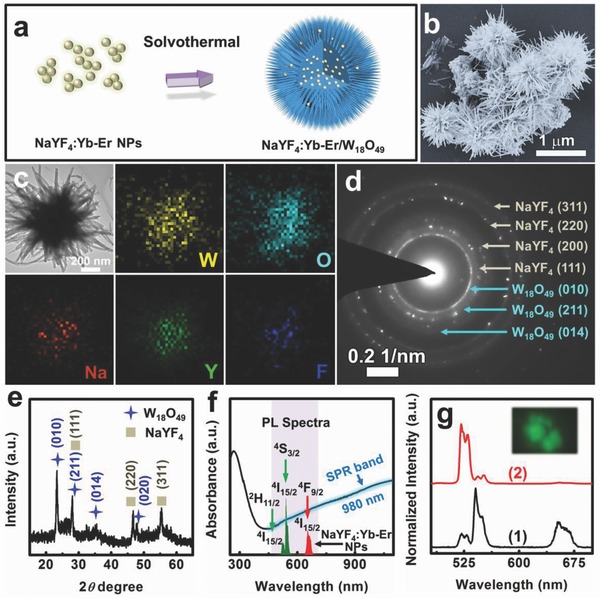
a) Schematic diagram of the synthesis route for the NaYF_4_:Yb‐Er@W_18_O_49_ quasi‐core/shell heterostructure; b) SEM image of the NaYF_4_:Yb‐Er@W_18_O_49_ heterostructures; c) TEM image and the corresponding elemental mapping of an individual NaYF_4_:Yb‐Er@W_18_O_49_ heterostructure; d) SAED and e) XRD patterns of the NaYF_4_:Yb‐Er@W_18_O_49_ heterostructures; f) UV–vis–NIR absorption spectra of the NaYF_4_:Yb‐Er@W_18_O_49_ heterostructures and the upconversion emission spectra of the NaYF_4_:Yb‐Er NPs; g) normalized upconversion emission spectra of 1) the NaYF_4_:Yb‐Er NPs and 2) the NaYF_4_:Yb‐Er@W_18_O_49_ heterostructures. The inset provides the micro‐area optical image of the 980 nm excited NaYF_4_:Yb‐Er@W_18_O_49_ heterostructures.

The catalytic activity of the as‐synthesized NaYF_4_:Yb‐Er@W_18_O_49_ heterostructure was evaluated via hydrolysis of BH_3_NH_3_ under irradiation of 980 nm laser diode with a spot area of ≈0.5 cm^2^. As shown in **Figure** **5**a, in the absence of catalysts, a very low H_2_ evolution happened after 1 h of 980 nm irradiation (≈0.06 µmol), suggesting a slow hydrolysis process of BH_3_NH_3_. When adding W_18_O_49_ NWs into the reaction solution without light irradiation, a slight increase in the H_2_ evolution (≈0.12 µmol) could be observed (Figure S12, Supporting Information), demonstrating the poor catalytic activity of unexcited plasmonic W_18_O_49_ NWs. However, upon 980 nm irradiation, the H_2_ evolution amount was substantially increased to ≈0.60 µmol after 1 h. The pure NaYF_4_:Yb‐Er NPs showed a negligible H_2_ generation (Figure S12, Supporting Information). These results revealed that the excited LSPR of W_18_O_49_ NWs could greatly boost the catalytic H_2_ evolution due to the plasmonic transfer process of “hot electrons,” as outlined in previous publications.^21^, ^27^ Importantly, the H_2_ evolution amount of the NaYF_4_:Yb‐Er@W_18_O_49_ heterostructure can reach ≈2.11 µmol with the apparent quantum efficiency of ≈2.8% under 1 h irradiation of 980 nm (Equation (S22), Supporting Information). This H_2_ evolution amount is about 3.5 times higher than the H_2_ evolution of pure W_18_O_49_ NWs and even 35 times higher than the H_2_ evolution of single BH_3_NH_3_ hydrolysis. Meanwhile, the apparent quantum efficiency is comparable to the corresponding values obtained in other visible‐light photocatalysts.^40^, ^41^, ^42^ This implies that the upconversion NPs play a central role on the enhanced catalytic activity of the H_2_ evolution. Namely, the upconversion emission of Ln^3+^‐doped NaYF_4_ NPs at a suitable wavelength region can improve the LSPR excitation of the W_18_O_49_ NWs and enhance the catalytic activity for H_2_ evolution on the surface of W_18_O_49_ NWs. This hypothesis can be verified via wavelength‐depended H_2_ evolution plots of W_18_O_49_ NWs. As shown in Figure 5b, the H_2_ evolution amount of W_18_O_49_ NWs is directly correlated with the photon energy that can be absorbed by the W_18_O_49_ NWs to drive the LSPR effect.^21^, ^24^, ^27^, ^38^ It is apparent that the LSPR band of W_18_O_49_ NWs overlapped with the green and red emissions of the NaYF_4_:Yb‐Er NPs. As a result of this, the enhanced catalytic activity of the NaYF_4_@W_18_O_49_ heterostructures can be attributed to the NIR‐plasmonic energy upconversion induced by the LSPR effect of W_18_O_49_ NWs.

**Figure 5:**
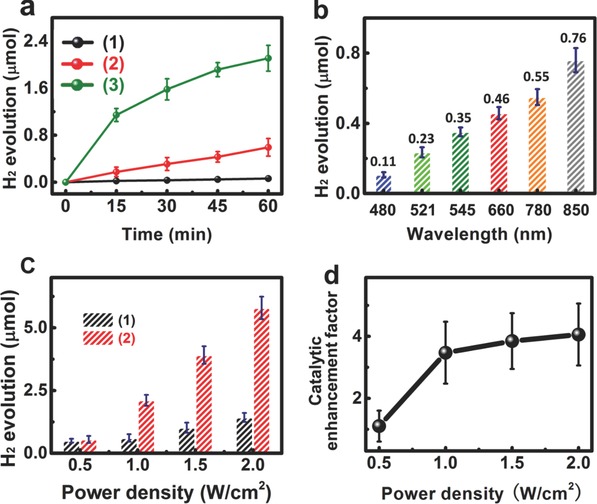
a) Plots of H_2_ evolution amount versus reaction time of different catalysts in the BH_3_NH_3_ aqueous solution under irradiation at 980 nm: 1) Without any catalyst; 2) W_18_O_49_ NWs; 3) NaYF_4_:Yb‐Er@W_18_O_49_ heterostructures; b) H_2_ evolution amount of the W_18_O_49_ NWs under irradiation with different incident light wavelengths for 1 h; c) power density‐dependent H_2_ evolution amount of 1) W_18_O_49_ NWs and 2) NaYF_4_:Yb‐Er@W_18_O_49_ heterostructures; d) plot of enhancement factor versus laser power density over NaYF_4_:Yb‐Er@W_18_O_49_ heterostructures.

If this were true, the catalytic enhancement factor should be related to the excitation power density of the incident light.^43^, ^44^ As shown in Figure 5c, the H_2_ evolution amounts of both the W_18_O_49_ NWs and NaYF_4_:Yb‐Er@W_18_O_49_ heterostructures depended strongly on the power density of 980 nm laser diode. Furthermore, the enhancement factor gradually increased with the power density of incident light (Figure 5d). Therefore, it can be concluded that the light intensity illuminated on W_18_O_49_ dominated the enhanced catalytic activity. Besides, the NaYF_4_@W_18_O_49_ heterostructure was also a reusable plasmonic catalyst (Figure S13, Supporting Information). Furthermore, the catalytic activity of NaYF_4_:Yb‐Er@W_18_O_49_ heterostructures for H_2_ evolution increases with the size of NaYF_4_:Yb‐Er hetero‐component (Figure S14, Supporting Information). Also note that a NaYF_4_:Yb‐Er/W_18_O_49_ film with the larger sizes of NaYF_4_:Yb‐Er NPs exhibited a higher intensity of upconversion luminescence, but a smaller enhancement factor. Thus, coupling plasmonic W_18_O_49_ NWs with the larger sizes of NaYF_4_:Yb‐Er NPs in their heterostructure could lead to a more efficient NIR‐plasmonic energy upconversion to excite the LSPR of W_18_O_49_ NWs for catalytic H_2_ evolution.

In summary, we have demonstrated the selective enhancement of upconversion luminescence behavior on the well‐designed heterostructure film fabricated via self‐assembly of NaYF_4_:Yb‐Er NPs onto the nonmetallic plasmonic layer of W_18_O_49_ NWs grown on an FTO glass substrate. Investigations showed that the broad LSPR absorption band of W_18_O_49_ NWs overlapped with both the excitation and emission bands of NaYF_4_:Yb‐Er NPs, which could induce an NIR‐plasmonic energy upconversion process, thus resulting in 9‐ to 112‐fold enhancement of green emission on the NaYF_4_:Yb‐Er/W_18_O_49_ film as compared to the pure NaYF_4_:Yb‐Er NP film. By using this fascinating photophysical process, we also realized a promoted catalytic activity for H_2_ evolution from BH_3_NH_3_ over the quasi‐core/shell heterostructure of NaYF_4_:Yb‐Er NPs@W_18_O_49_ NWs under low‐energy 980 nm excitation. The amount of H_2_ evolution of the heterostructure was 35 times higher than that of BH_3_NH_3_ hydrolysis after irradiation with 980 nm for 1 h. Our study not only developed feasible and low‐cost tactics to selectively enhance the upconversion luminescence of Ln^3+^‐doped NaYF_4_ NPs, but also offered a new class of NIR‐responsive heterostructure catalysts that possess an efficient catalytic activity for H_2_ evolution based on the NIR‐plasmonic energy upconversion process.

## Conflict of Interest

The authors declare no conflict of interest.

## Supporting information

Supplementary
