# Supplementary material for: Near‐Infrared‐Plasmonic Energy Upconversion in a Nonmetallic Heterostructure for Efficient H2 Evolution from Ammonia Borane
Source: Adv Sci (Weinh). 2018 Jul 3;5(9):1800748. doi: 10.1002/advs.201800748 (PMC6145233; doi:10.1002/advs.201800748)
Supplement: Supplementary file 1 — Supplementary [file ADVS-5-1800748-s001.pdf]

Copyright WILEY-VCH Verlag GmbH & Co. KGaA, 69469 Weinheim, Germany, 2018.

ADVANCED SCIENCE
Open Access

Supporting Information

for *Adv. Sci.,* DOI: 10.1002/advs.201800748

NIR-Plasmonic Energy Upconversion in a Nonmetallic Heterostructure for Efficient H_2 Evolution from Ammonia Borane

*Zhenyi Zhang, Yang Liu, Yurui Fang, Baosheng Cao, Jindou Huang, Kuichao Liu, and Bin Dong**

Copyright WILEY-VCH Verlag GmbH & Co. KGaA, 69469 Weinheim, Germany, 2016.

Supporting Information

NIR-Plasmonic Energy Upconversion in a Nonmetallic Heterostructure for Efficient H_2 Evolution from Ammonia Borane

Zhenyi Zhang[1], Yang Liu[1,3], Yurui Fang[2], Baosheng Cao[1], Jindou Huang[1], Kuichao Liu[1] & Bin Dong[1,]*

[1] Key Laboratory of New Energy and Rare Earth Resource Utilization of State Ethnic Affairs Commission, Key Laboratory of Photosensitive Materials & Devices of Liaoning Province, School of Physics and Materials Engineering, Dalian Nationalities University, 18 Liaohe West Road, Dalian 116600, P. R. China

[2] Key laboratory of Materials Modification by Laser, Electron, and Ion Beams (Ministry of Education), School of Physics, Dalian University of Technology, Dalian 116024, P. R. China

[3] School of Materials and Engineering, Dalian University of Technology, Dalian 116024, P. R. China
*Corresponding author: dong@dlnu.edu.cn

Experimental Section

(1) Synthesis of Upconversion Luminescence Nanoparticles (NPs): 0.78 mmol of $YCl_3·6H_2O$, 0.20 mmol of $YbCl_3·6H_2O$, and 0.02 mmol of $ErCl_3·6H_2O$ were mixed with a solution consisting of 6 mL oleic acid and 15 mL octadecene in a 100 mL flask, which was then heated to 160°C for 30 min under N_2 gas protection to form a homogeneous solution. Subsequently, this solution was cooled to room temperature at a flow of N_2 gas though the reaction flask. During this process, 10 mL of methanol solution containing NaOH (2.5 mmol) and NH_4F (4 mmol) was slowly dropped into the reaction flask. The obtained solution was stirred for 30 min. Subsequently, the reaction temperature was increased to 80°C to evaporate methanol from the reaction solution; in succession, the solution was heated to 300°C where it was maintained for 1 h under N_2 atmosphere. When the solution had naturally cooled down, the products of $NaYF_4$:Yb-Er NPs with mean sizes of 40 nm were collected via centrifugation, and washed with ethanol/cyclohexane (3:1 v/v) thrice and finally dispersed in cyclohexane solvent with a concentration of ~0.1 M. The $NaYF_4$:Yb-Er NPs with mean sizes of 10 nm and 20 nm were obtained through controlling the reaction temperature at 280 and 290°C, respectively. Please note that when the reaction temperature was reduced to 280°C, the 10-nm $NaYF_4$:Yb-Er NPs could be obtained while their phase structures changed from β to α.

(2) Fabrication of films of plasmonic $W_{18}O_{49}$ Nanowires (NWs) on FTO glass: In a typical process, 25 mg of $W(CO)_6$ was dissolved into 20 mL of absolute ethanol under constant stirring to form a yellow transparent solution. Then, a cleansed FTO glass at a size of 2 cm × 3 cm was placed into a Teflon-lined autoclave, loaded with the above solution. The autoclave was sealed and then maintained at 180°C for 12 h. The obtained sample on the FTO glass with blue color was removed from the reaction solution, washed with ethanol, and finally dried in nitrogen.

(3) Fabrication of $NaYF_4/W_{18}O_{49}$ films: In a typical process, 0.1 mL of $NaYF_4$:Yb-Er NPs-suspended cyclohexane solution (0.1 M) was dropped into 10 mL of cyclohexane solution under ultrasonic treatment for 20 min. After that, the FTO glass loaded with plasmonic $W_{18}O_{49}$ NWs film was vertically immersed into the above suspended solution and then put in an oven at 35°C for 2 h. With the slow evaporation of ethanol solvent, a thin film consisting of $NaYF_4$:Yb-Er NPs laxly self-assembled onto the surface of $W_{18}O_{49}$ NWs grown on the FTO glass. Based on a similar fabrication method, two control samples were also constructed on the FTO glass: one was the individual $NaYF_4$:Yb-Er film that was obtained via direct self-assembly of the $NaYF_4$:Yb-Er NPs onto the FTO glass; the other is the $NaYF_4$:Yb-Er/N-$W_{18}O_{49}$ film where the N-$W_{18}O_{49}$ NWs, denoting the non-plasmonic $W_{18}O_{49}$ NWs, was obtained via H_2O_2-treatment of plasmonic $W_{18}O_{49}$ NWs to passivate their surface oxygen vacancies and thus to quench the LSPR absorption feature (Figure S1).

(4) Synthesis of $NaYF_4$:Yb-Er@$W_{18}O_{49}$ quasi-core/shell heterostructures: For the first step, 1.2 mmol of NaCl, 0.48 mmol of YCl_3, 0.108 mmol of $YbCl_3$, and 0.012 mmol of $ErCl_3$ were mixed in 9 mL of ethylene glycol (EG) solvent to form a transparent solution that was labeled as solution A. Concurrently, 3.0 mmol of NH_4F and 0.006 mmol of polyethyleneimine were dissolved into 6 mL of EG solvent to obtain the other transparent solution that was labeled solution B. The resulting solutions A and B were then mixed and agitated for 10 min, transferred into a 25 mL of Teflon-lined autoclave, and kept at 200°C for 2 h. The products of hydrophilic α-$NaYF_4$:Yb-Er NPs with a mean size of 40 nm were collected via centrifugation, washed with ethanol four times and finally dispersed in an ethanol solvent at a concentration of ~0.1 M; For the second step, 25 mg of $W(CO)_6$ was dissolved into 20 mL of absolute ethanol under constant stirring. Then, 50 μL of the α-$NaYF_4$:Yb-Er NPs(40 nm)-suspended

ethanol solution (0.1 M) was gradually dropped in the above solution and sealed in a Teflon-lined autoclave. The autoclave was maintained at 180°C for 12 h. The precipitates with blue color were collected via centrifugation, washed with ethanol four times, and finally dried in vacuum.

Catalytic H_2 evolution:

2 mg of the as-synthesized catalysts were dispersed into 4 mL of deionized water (Scheme S1). The obtained suspension solution was placed in a photoreactor with a volume of 35 mL under constant stirring. The reactor was then sealed and degassed with argon for 10 min. Subsequently, 2 mL of deionized water containing 2 mg of ammoniaborane was injected into the photoreactor, which was exposed to 980-nm laser diode or a 300 W Xe lamp (PLS-SXE300UV) coupled with a monochromator (The intensity of monochromatic light at ~8 mw/cm^2). The surrounding temperature was fixed at 28℃ by using a connected reflux water condenser. The generated H_2 was periodically analyzed via gas chromatograph, equipped with a thermal conductivity detector (Beifen-Ruili Analytical Instrument, SP-3420A).

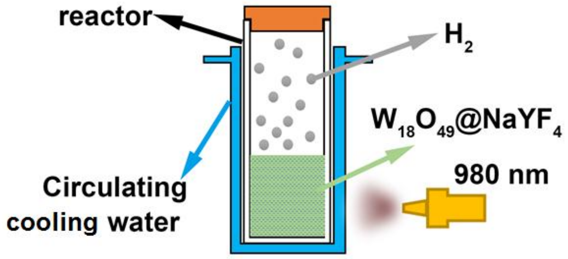

Scheme S1 Schematic illustration of the photocatalytic experimental setup.

Characterization

X-ray diffraction (XRD) patterns of the as-synthesized samples were recorded via Shimadzu XRD-6000 X-ray diffractometer with a Cu Kα line of 0.1541 nm. Scanning electron microscopy (SEM; XL-30 ESEM FEG, Micro FEI Philips) and transmission electron microscopy (TEM; JEOL JEM-2100) were used to investigate the morphologies and structures of samples. The UV–vis absorption spectra of the samples were recorded on a Lambda 750 UV–Vis–NIR spectrophotometer (Perkin-Elmer, USA). The upconversion emission properties of products were measured with an inverted microscope (Olympus IX71) combined with a spectrometer (PI Instrument). Excitation with a 980-nm laser passing a laser clean-up was reflected into the objective (50X Olympus) via a dichroic short pass filter. The upconversion emission was collected with the same objective and conducted into the spectrometer. The laser clean-up and dichroic filter was used to purify the excitation light and to eliminate the laser line before the emission was detected (Scheme 1).

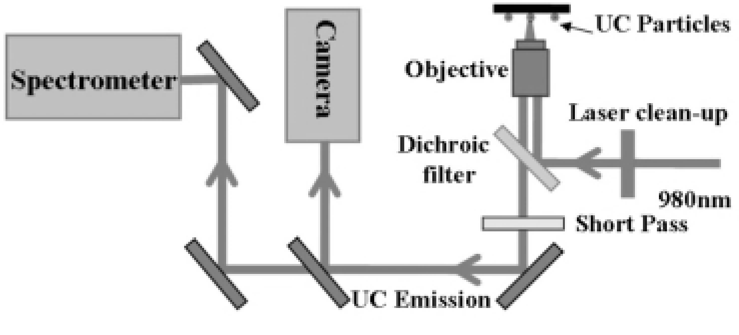

Scheme S2 Schematic illustration of the set-up used to measure the upconversion emissions of as-fabricated products.

Finite element method (FEM) simulation

All full wave numerical simulations were performed with the finite element method (FEM, commercial software package, Comsol Multiphysics 5.0). The $W_{18}O_{49}$ (permittivity was obtained with the following simulation. See scheme S2) nanowire (diameter D_1 = 10 nm, length L = 800 nm) bundle (three wires touching each other) was placed in a homogeneous surrounding medium with an effective refractive index of 1.0. $NaYF_4$ sphere (permittivity ε = 2.477 at 980 nm, diameter D_2 = 40 nm) was put in direct contact with the $W_{18}O_{49}$ nanowire at different positions. Non-uniform meshes were used to format the object. The largest mesh was set to below $\lambda/6$. A perfect matched layer (PML) was used to minimize scattering from the outer boundary. The structure was placed in the x-y plane. The incident light was set to 1 V/m polarized in y-direction and propagated in z-direction.

Optical property simulation for $W_{18}O_{49}$

The Cambridge Serial Total Energy Package (CASTEP) has been used for optical property calculations, which is based on the density functional theory (DFT) and utilizes a plane-wave pseudopotential method.[1] We used the generalized gradient approximation (GGA) in the scheme of Perdew–Burke–Ernzerhof (PBE) to describe the exchange-correlation functional.[2] The interaction between valence electrons and the ionic core was described via ultrasoft pseudopotential.[3] An energy cutoff of 300 eV was chosen for the $W_{18}O_{49}$ crystal. The Brillouin-zone sampling mesh parameters for the *k*-point set were 2×2×2 for 67 atoms systems. In the optimization process, the energy change, maximum force, maximum stress, and maximum displacement tolerances were set as 2×10^{-6} eV/atom, 0.05 eV/Å, 0.1 Ga, and 0.002 Å, respectively.

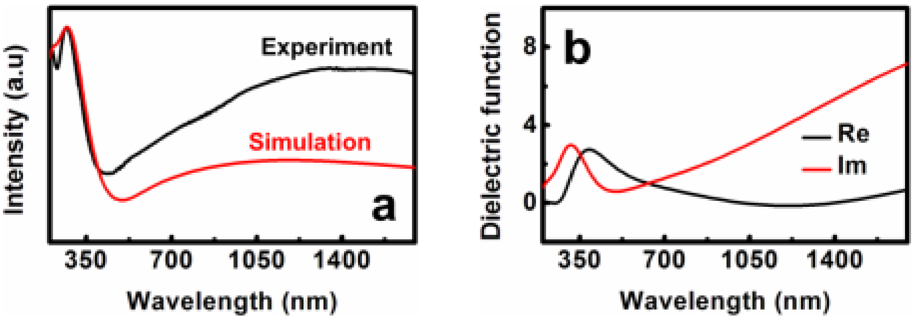

Scheme S3 (a) UV-Vis-NIR absorption spectra of the $W_{18}O_{49}$ WNs obtained via simulation and experiment; (b) simulated dielectric function of $W_{18}O_{49}$ WNs.

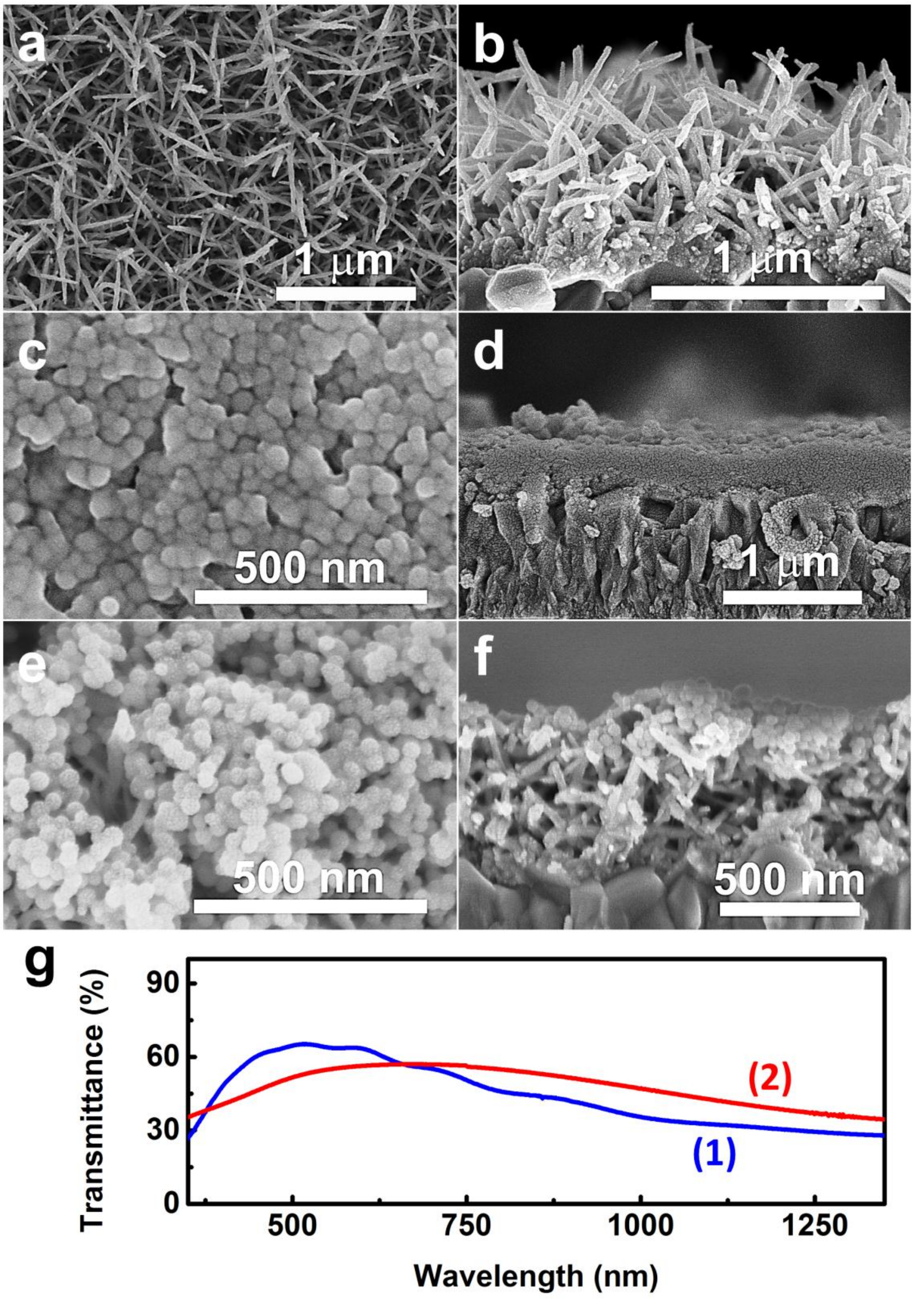

Figure S1 SEM images of the top views and side views of (a, b) $W_{18}O_{49}$ WNs, (c, d) $NaYF_4$:Yb-Er NPs (40 nm), and (e, f) $NaYF_4$:Yb-Er(40 nm)/N-$W_{18}O_{49}$ film on the FTO glass, respectively; (g) Comparaison transmittance spectra of $W_{18}O_{49}$ WNs film positioned at room temperature and atmosphere pressure for (1) 0 day and (2) 90 days.

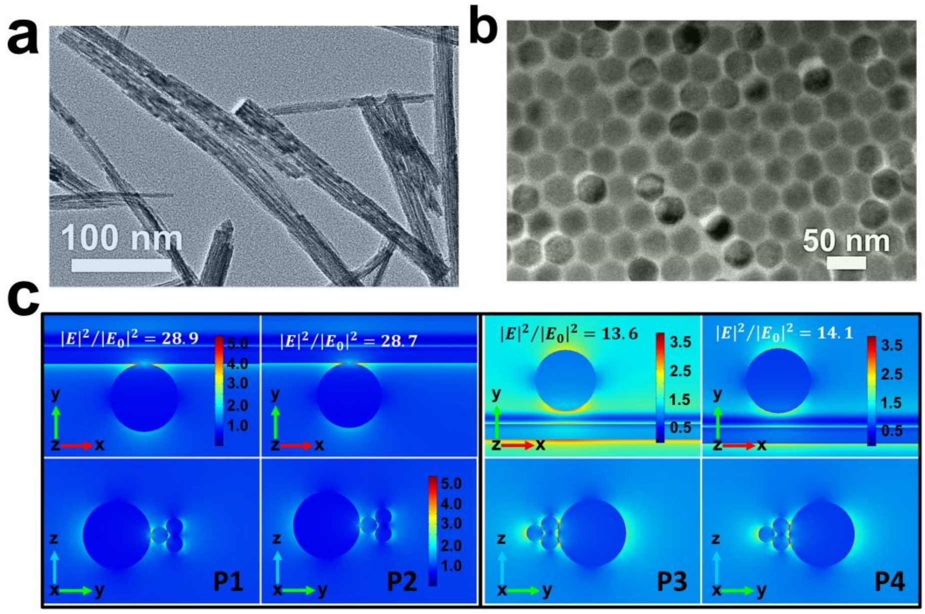

Figure S2 TEM images of (a) $W_{18}O_{49}$ NWs and (b) $NaYF_4$:Yb-Er NPs; (c) simulations of excitation electric field distribution induced via plasmonic $W_{18}O_{49}$ NWs and extending to the adjacent $NaYF_4$:Yb-Er NPs with input irradiation along the y-axis: the $NaYF_4$:Yb-Er NP loaded on the representative positions of P1, P2, P3 or P4.

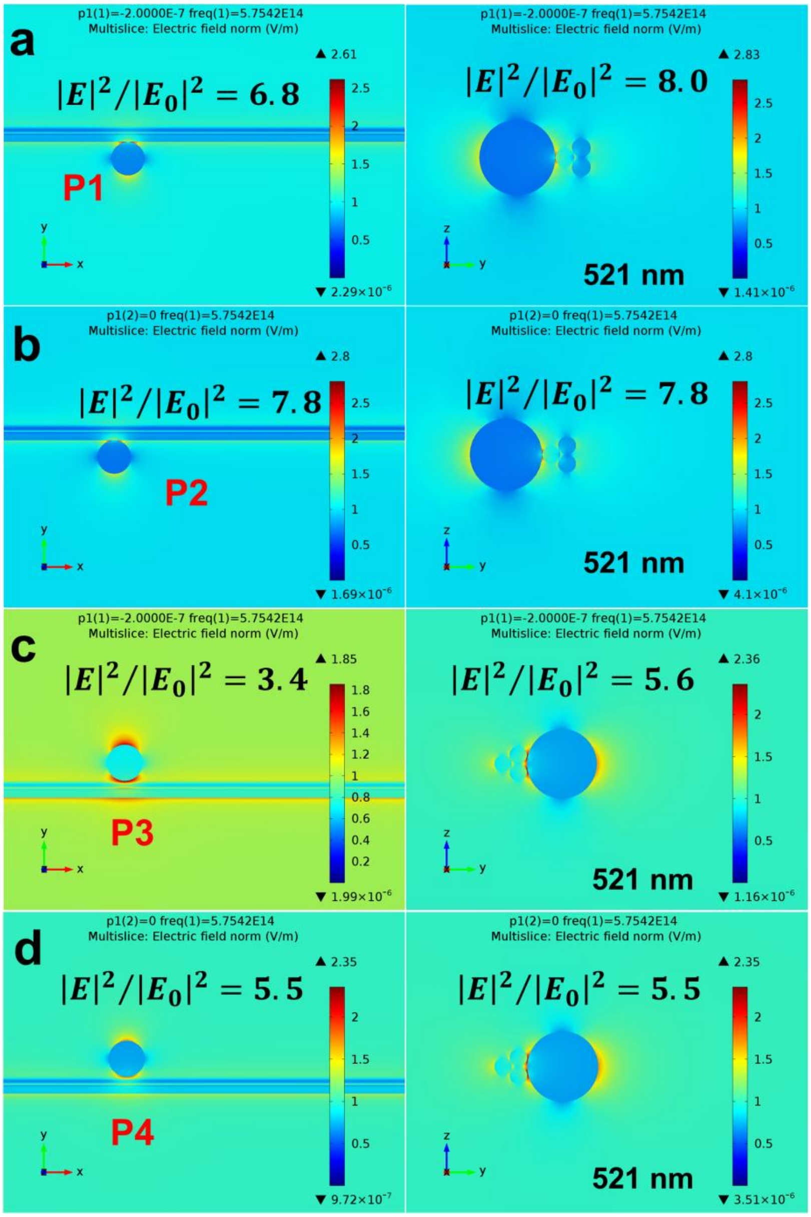

Figure S3 FEM simulation of the emission electric field distribution (521 nm) induced via plasmonic $W_{18}O_{49}$ NWs after 980-nm excitation and extending to the adjacent $NaYF_4$ NPs (size: 40 nm) with input irradiation along the y-axis: the $NaYF_4$ NP was loaded on the representative positions of (a) P1, (b) P2, (c) P3, or (d) P4.

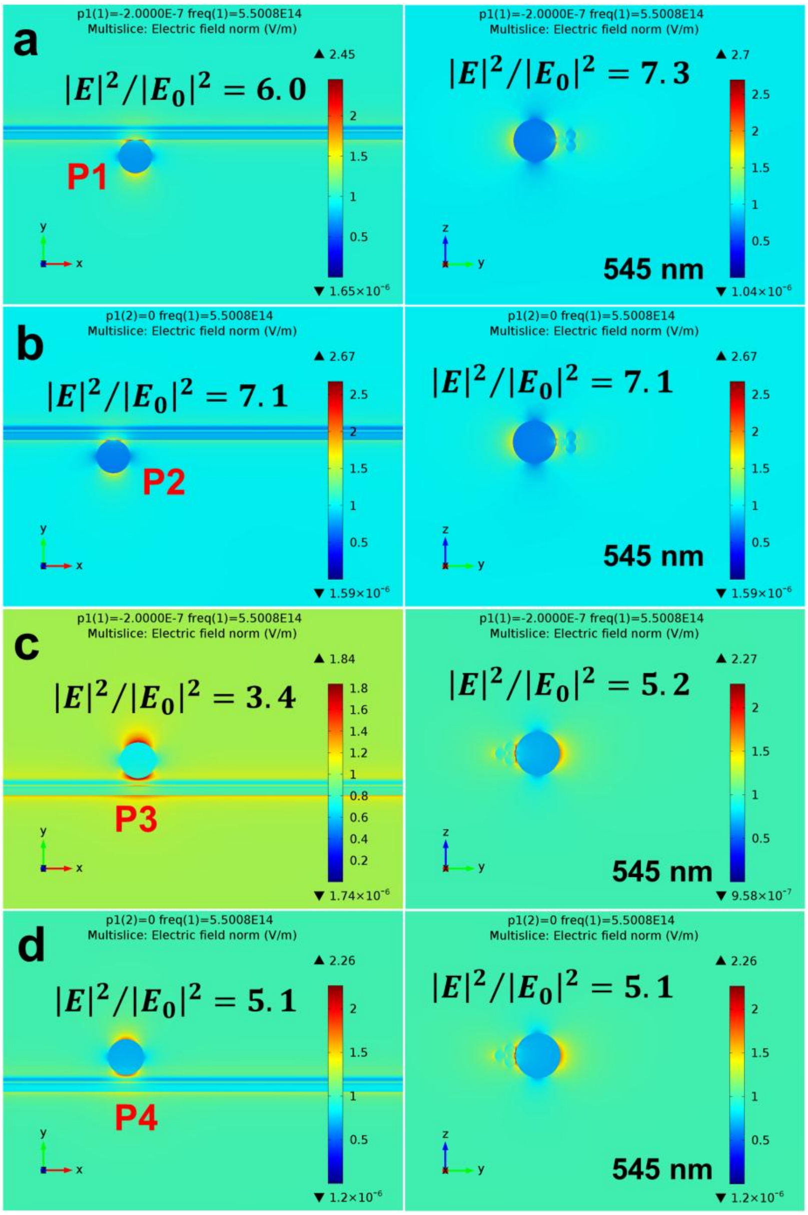

Figure S4 FEM simulation of emission electric field distribution (545 nm) induced via plasmonic $W_{18}O_{49}$ NWs after 980-nm excitation and extending to the adjacent $NaYF_4$ NPs (size: 40 nm) with input irradiation along the y-axis: the $NaYF_4$ NP was loaded on the representative positions of (a) P1, (b) P2, (c) P3, or (d) P4.

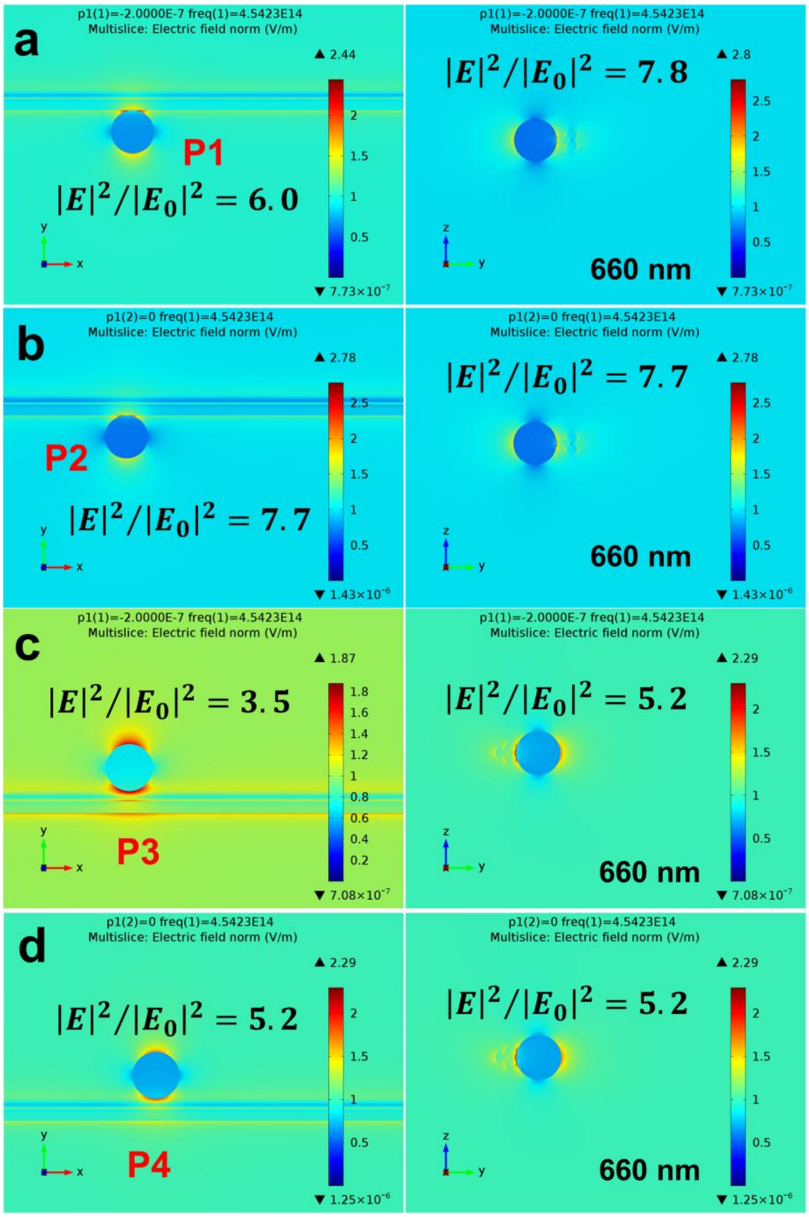

Figure S5 FEM simulation of the emission electric field distribution (660 nm) induced by the plasmonic $W_{18}O_{49}$ NWs after 980-nm excitation and extending to the adjacent $NaYF_4$ NPs (size: 40 nm) with input irradiation along the y-axis: the $NaYF_4$ NP was loaded on the representative positions of (a) P1, (b) P2, (c) P3, or (d) P4.

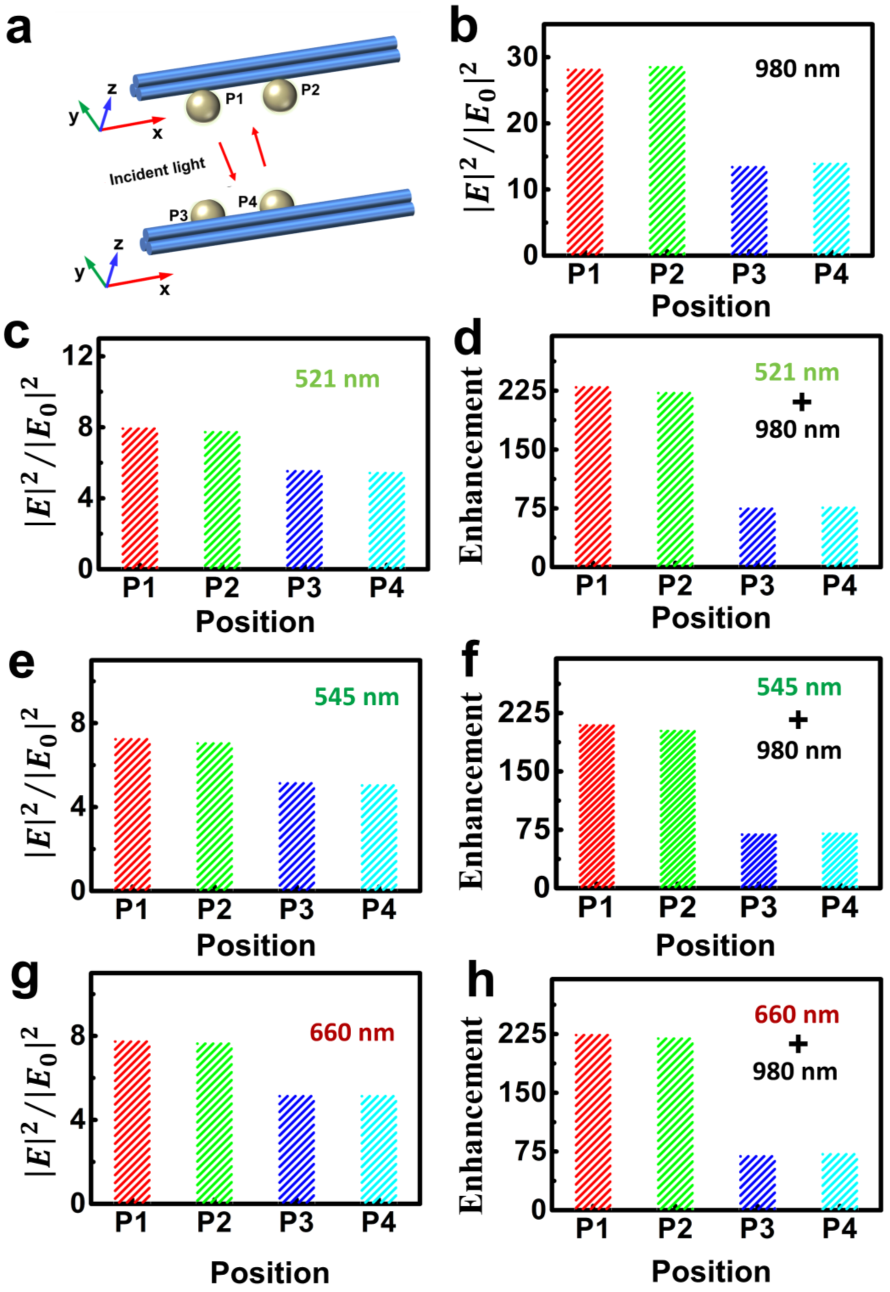

Figure S6 (a) Simulation model of the $NaYF_4/W_{18}O_{49}$ heterostructure with the $NaYF_4$ NPs (size: 40 nm) loaded on the representative positions of the $W_{18}O_{49}$ NWs surface; (b) excitation electric field intensity enhancement (980 nm) at the plasmonic "hot spots" as a function of the contact positions between the $NaYF_4$ NPs and $W_{18}O_{49}$ NWs; emission electric field intensity enhancement at the plasmonic "hot spots" as a function of the contact positions between the $NaYF_4$ NPs and $W_{18}O_{49}$ NWs: (c) 521 nm, (e) 545 nm, and (g) 660 nm; enhancement of the overall electric field intensity at the plasmonic "hot spots" as a function of the contact positions between the $NaYF_4$ NPs and $W_{18}O_{49}$ NWs: (d) excitation at 980 nm and emission at 521 nm, (f) excitation at 980 nm and emission at 545, and (h) excitation at 980 nm and emission at 660 nm.

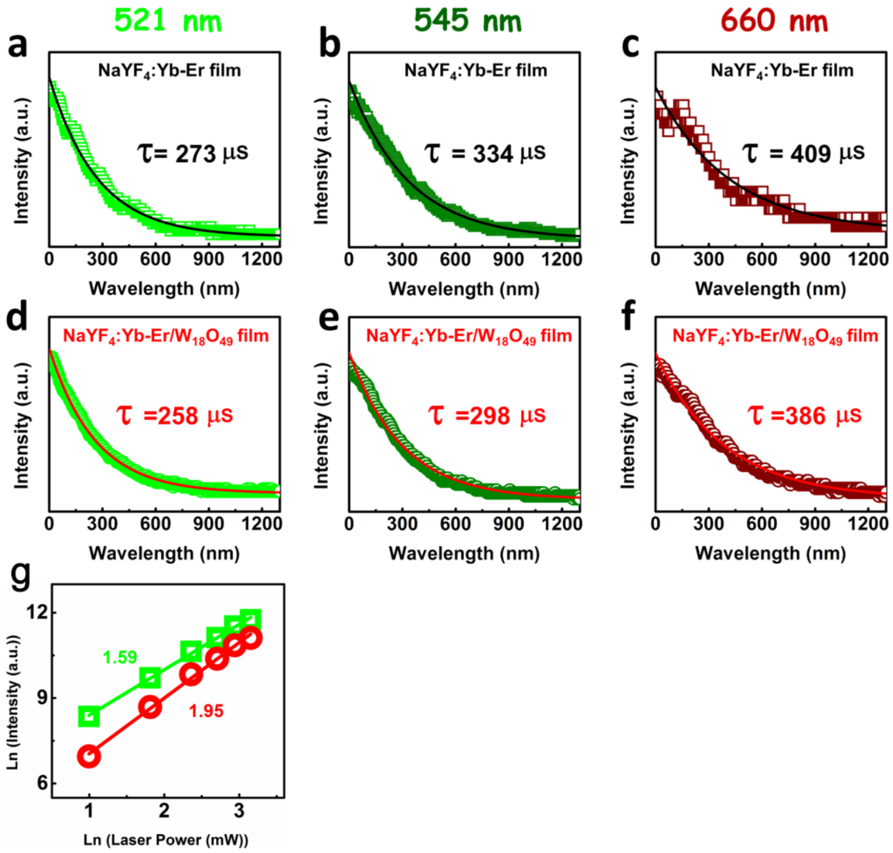

Figure S7 (a-f) Time-resolved decay curves of $^2I_{11/2}$→$^4I_{15/2}$ (521 nm), $^4S_{3/2}$→$^4I_{15/2}$ (545 nm), and $^4F_{9/2}$-$^4I_{15/2}$ (660 nm) transitions in $NaYF_4$:Yb-Er and $NaYF_4$:Yb-Er/$W_{18}O_{49}$ films, respectively, under 980-nm excitation; (g) Pump power dependence of upconversion emission intensities of $NaYF_4$:Yb-Er NPs.

We carried out the time-resolved luminescence spectroscopy measurements, which is particularly useful for probing the actual influence of emission-matched plasmonic nanostructures on upconversion NPs. In our case, the lifetimes of $^2I_{11/2}$→$^4I_{15/2}$ (521 nm), $^4S_{3/2}$→$^4I_{15/2}$ (545 nm), and $^4F_{9/2}$-$^4I_{15/2}$ (660 nm) decays for the $NaYF_4$:Yb-Er/$W_{18}O_{49}$ film were shorter than the corresponding lifetimes obtained from the individual $NaYF_4$:Yb-Er film (Figure S6 a-f). However, the comparison of upconversion luminescence spectra between the $NaYF_4$:Yb-Er and $NaYF_4$:Yb-Er/$W_{18}O_{49}$ films indicated that the introduction of $W_{18}O_{49}$ NWs into the $NaYF_4$:Yb-Er film could lead to a remarkable enhancement of green emission from the $^2I_{11/2}$→$^4I_{15/2}$ (521 nm) transition, but the quenched emissions from both $^4S_{3/2}$→$^4I_{15/2}$ (545 nm) and $^4F_{9/2}$-$^4I_{15/2}$ (660 nm) transitions. A decrease in decay lifetime coincident with an increase in emission suggests the enhancement of radiative rate of the $^2I_{11/2}$→$^4I_{15/2}$ (521 nm) transition. On the other hand, the decreased decay lifetime and emission quenching indicate the enhanced non-radiative decay process for the $^4S_{3/2}$→$^4I_{15/2}$ (545 nm) and $^4F_{9/2}$-$^4I_{15/2}$ (660 nm) transitions.[4] These results reveal the existence of plasmon-mediated competition between the radiative and non-radiative processes in the $NaYF_4$:Yb-Er/$W_{18}O_{49}$ film after 980-nm excitation. Figure S6g shows the pump power dependence of red and green upconversion emission intensity of $NaYF_4$:Yb-Er NPs. After a linear fitting analysis, we found that the slopes are 1.95 and 1.59 for red and green emissions. The values are ~2, indicating the two-photon required for the upconversion process.

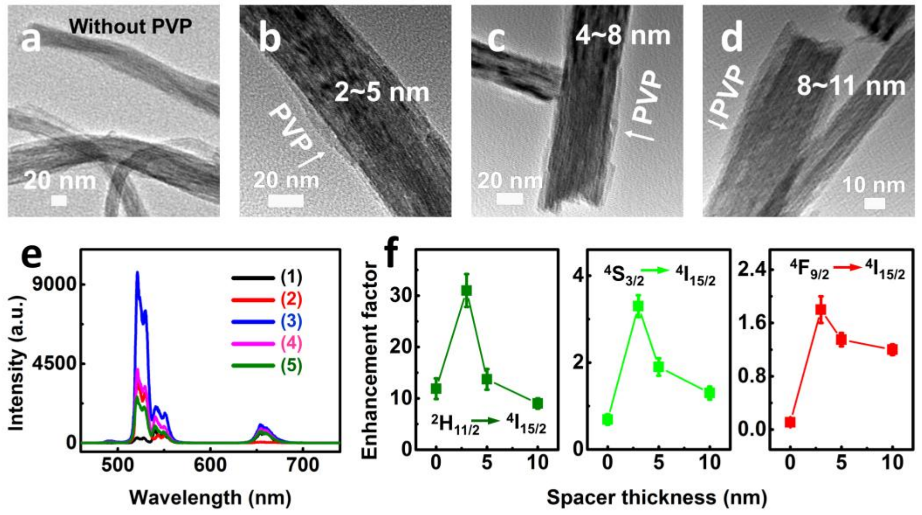

Figure S8 TEM images of $W_{18}O_{49}$ NWs treated by PVP solutions with the concentration of (a) 0 mg/mL, (b) 3 mg/mL, (c) 6 mg/mL, and (d) 10 mg/mL; (e) upconversion emission spectra of (1) $NaYF_4$:Yb-Er film, (2) $NaYF_4$:Yb-Er/$W_{18}O_{49}$ film, and the $NaYF_4$:Yb-Er/PVP/$W_{18}O_{49}$ film with the PVP thicknesses of (3) 2~5 nm, (4) 4~8 nm, and (5) 8~11 nm; (f) enhancement factor of upconversion luminescence as a function of the thickness of PVP spacer in the $NaYF_4$:Yb-Er/PVP/$W_{18}O_{49}$ film.

Here, we employed the polyvinylpyrrolidone (PVP, Mw=1300 000) polymer as the insulating spacer to separate $NaYF_4$:Yb-Er and $W_{18}O_{49}$ components in the composite film. The thickness of PVP spacer on the $W_{18}O_{49}$ surface was controlled through adjusting the concentration of PVP solution during the impregnation process. To confirm the thicknesses of PVP spacers on the surfaces of $W_{18}O_{49}$ NWs, the PVP-covered $W_{18}O_{49}$ NWs were exfoliated from the FTO film to observe under TEM. As shown in Figure S7 a-d, with the increase of PVP concentration from 0 to 10 mg/mL, the thicknesses of PVP spacers increase gradually from ~2 to ~11 nm on the surfaces of $W_{18}O_{49}$ NWs. Meanwhile, the properties of upconversion luminescence for these films change significantly (Figure S7 e). When the PVP spacers were 2~5 nm in thicknesses, the upconversion luminescence of $NaYF_4$:Yb-Er/PVP/$W_{18}O_{49}$ film reached the optimal performance. The emission intensities of $NaYF_4$:Yb-Er/PVP/$W_{18}O_{49}$ film from $^2I_{11/2}$→$^4I_{15/2}$ (521 nm), $^4S_{3/2}$→$^4I_{15/2}$ (545 nm), and $^4F_{9/2}$-$^4I_{15/2}$ (660 nm) transitions were 30, 3.3, and 1.8 times higher than the corresponding values of the individual $NaYF_4$:Yb-Er film (Figure S7 f). The enhancements on both red and green emissions suggest the weakened non-radiative energy transfer process in the $NaYF_4$:Yb-Er/PVP/$W_{18}O_{49}$ film. Thus, the LSPR-enhanced excitation and emission fields dominate the enhancement of upconversion luminescence for $NaYF_4$:Yb-Er NPs in the $NaYF_4$:Yb-Er/$W_{18}O_{49}$ film. However, when we further increased the thickness of PVP on the $W_{18}O_{49}$ surface, the enhancement effect on the upconversion luminescence was reduced due to the limited operating distance for the LSPR-enhanced near-filed effect.

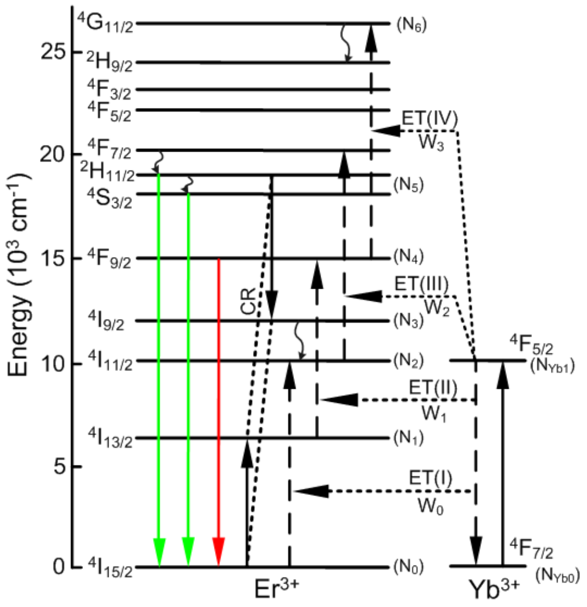

Figure S9 Energy levels of Yb^{3+} and Er^{3+} ions relevant to the energy-transfer upconversion process.

To understand qualitatively the photophysical mechanism in this plasmon-enhanced upconversion luminescence process, a set of rate equations were established based on the well -known upconversion process:

$$\frac{dN_1}{dt} = R'_{21}N_2 + C_{50}N_5N_0 - \sigma_{14} f^{ex} \rho N_1 - W_1 N_1 N_{Yb1} - R_1 N_1 \qquad \text{(S1)}$$

**Non-radiative decay from level 2 to 1 (negligible)**	**Cross-relaxation between level 5,3 and 0,1**	**Direct absorption of Er (negligible)**	**Energy transfer from level 1 to 4**	**Rediative decay from level 1 (not observed)**

$$\frac{dN_2}{dt} = \sigma_{02} f^{ex} \rho N_0 + R'_{32}N_3 + W_0 N_0 N_{Yb1} - \sigma_{25} f^{ex} \rho N_2 - W_2 N_2 N_{Yb1} - R_2 N_2 - R'_{21} N_2 \qquad \text{(S2)}$$

**Direct absorption of Er (negligible)**	**Non-radiative decay from level 3 to**	**Energy transfer from level 0 to 2**	**Direct absorption of Er (negligible)**	**Energy transfer from level 2 to 5**	**Radiative decay from level 2**	**Non-radiative decay from level 2 to 1 (negligible)**

$$\frac{dN_3}{dt} = R'_{43}N_4 + C_{50}N_5N_0 - R'_{32}N_3 - R_3 N_3 \qquad \text{(S3)}$$

**Non-radiative decay from level 4 to 3 (negligible)**	**Cross-relaxation between level 5,3 and 0,1**	**Non-radiative decay from level 3 to**	**Radiative decay from level 3**

$$\frac{dN_4}{dt} = R'_{54}N_5 + \sigma_{14} f^{ex} \rho N_1 + W_1 N_1 N_{Yb1} - \sigma_{46} f^{ex} \rho N_4 - W_3 N_4 N_{Yb1} - R'_{43} N_4 - R''_R N_4 - f_4^{em} R_4 N_4 \quad \text{(S4)}$$

Non-radia tive decay from level 5 to 4 (negligible	Direct absorpti on of Er (negligi ble)	Energy transfer from level 1 to 4	Direct absorpti on of Er (negligi ble)	Energy transfer from level 4 to 6 (negligible)	Non-radia tive decay from level 4 to 3 (negligible	Energy transfer from level 4 to $W_{18}O_{49}$	Radiative decay with emission field enhancement factor from level 4 (Red emission)

$$\frac{dN_5}{dt} = \sigma_{25} f^{ex} \rho N_2 + W_2 N_2 N_{Yb1} + R'_{65} N_6 - C_{50} N_5 N_0 - R'_{54} N_5 - R''_G N_5 - f_5^{em} R_5 N_5 \quad \text{(S5)}$$

Direct absorption of Er (negligible)	Energy transfer from level 2 to 5	Non-radi ative decay from level 6 to	Cross-rel axation between level 5,3 and 0,1	Non-radia tive decay from level 5 to 4 (negligible	Energy transfer from level 5 to $W_{18}O_{49}$	Radiative decay with emission field enhancement factor from level 5 (Green emission)

$$\frac{dN_6}{dt} = \sigma_{46} f^{ex} \rho N_4 + W_3 N_4 N_{Yb1} - R'_{65} N_6 - R_6 N_6 \quad \text{(S6)}$$

Direct absorpti on of Er (negligi ble)	Energy transfer from level 4 to 6	Non-radi ative decay from level 6 to	Radiative decay from level 6 (Blue

$$\frac{dN_{Yb1}}{dt} = \rho f^{ex} \sigma N_{Yb0} - W_0 N_0 N_{Yb1} - W_1 N_1 N_{Yb1} - W_2 N_2 N_{Yb1} - W_3 N_4 N_{Yb1} - R_{Yb1} N_{Yb1} \quad \text{(S7)}$$

Direct absorpti on of Yb	Energy transfer from level Yb1 to 0	Energy transfer from level Yb1 to 1 (lower N_{Yb1})	Energy transfer from level Yb1 to 2	Energy transfer from level Yb1 to 4	Radiative decay from level Yb1

$$N_{Er} = N_0 + N_1 + N_2 + N_3 + N_4 + N_5 + N_6 \quad \text{(S8)}$$

Population of level 0-6

$$N_{Yb} = N_{Yb0} + N_{Yb1} \quad \text{(S9)}$$

Population of level Yb0 and Yb1

Where N_0, N_1, N_2, N_3, N_4, N_5, and N_6 denote the population densities of $^4I_{15/2}$, $^4I_{13/2}$, $^4I_{11/2}$, $^4I_{9/2}$, $^4F_{9/2}$, ($^2H_{11/2}$+$^4S_{3/2}$), and $^4G_{11/2}$ levels of Er^{3+}, respectively. N_{Yb0}, and N_{Yb1} are the population densities of $^4F_{7/2}$ and $^4F_{5/2}$ levels of Yb^{3+}, respectively. N_{Er} and N_{Yb} are the nominal ions densities corresponding to Er and Yb codoping concentrations, respectively. R_1, R_2, R_3, R_4, R_5, and R_6 are the radiative rates of $^4I_{13/2}$, $^4I_{11/2}$, $^4I_{9/2}$, $^4F_{9/2}$, ($^2H_{11/2}$+$^4S_{3/2}$) and $^4G_{11/2}$ levels of Er^{3+}, respectively. R_{ij}' is non-radiative rate from level i to level j. R_G'' and R_R'' are the non-radiative energy transfer rates from the ($^2H_{11/2}$+$^4S_{3/2}$) and $^4F_{9/2}$ levels to the $W_{18}O_{49}$. W_i

(i=0-3) is the rate of the energy transfer process [ET(X) (x=I-IV)] from the excited Yb^{3+} to Er^{3+}. C_{50} is the cross relaxation for $^2H_{11/2}+^4I_{15/2}\rightarrow^2I_{13/2}+^4I_{9/2}$. ρ is the laser photon number density. f^{ex} is the enhancement factor of absorption (excitation field) caused by the $W_{18}O_{49}$'s LSPR. f_4^{em} and f_5^{em} are the enhancement factors of red and green radiative processes (emission field) caused by the $W_{18}O_{49}$'s LSPR, respectively. σ_{ij} denotes the absorption cross-section between level i and j of Er^{3+}, while σ is the absorption cross-section of Yb^{3+}. Taking into account that the excited state populations (<10^{16} ions/cm^3) are a small fraction of all the Er^{3+} or Yb^{3+} ions (N_{Er}, N_{Yb}>10^{20} ions/cm^3), it can be assumed that $N_0\approx N_{Er}$ and $N_{Yb0}\approx N_{Yb}$. At steady state (dN_i/dt=0), the rate equations can be simplified as:

$$C_{50}N_5N_0 - W_1N_1N_{Yb1} = 0 \quad \text{(S10)}$$

$$R'_{32}N_3 + W_0N_0N_{Yb1} - W_2N_2N_{Yb1} - R_2N_2 = 0 \quad \text{(S11)}$$

$$C_{50}N_5N_0 - R'_{32}N_3 = 0 \quad \text{(S12)}$$

$$W_1N_1N_{Yb1} - R''_RN_4 - f_4^{em}R_4N_4 = 0 \quad \text{(S13)}$$

$$W_2N_2N_{Yb1} - C_{50}N_5N_0 - R''_GN_5 - f_5^{em}R_5N_5 = 0 \quad \text{(S14)}$$

$$\rho f^{ex}\sigma N_{Yb0} - W_0N_0N_{Yb1} = 0 \quad \text{(S15)}$$

$$N_{Er} = N_0 \quad \text{(S16)}$$

$$N_{Yb} = N_{Yb0} \quad \text{(S17)}$$

The intensities of red (I_{Red}) and green (I_{Green}) emissions can be given according to the population densities of N_4 and N_5, respectively:

$$I_{Red} = f_4^{em}R_4N_4 = \frac{C_{50}N_{Er}N_{Yb}^2W_2\left(f^{ex}\right)^2\rho^2\sigma^2}{\left(1+\frac{R''_R}{f_4^{em}R_4}\right)\left[N_{Er}R_2\left(f_5^{em}R_5+R''_G\right)W_0 + C_{50}N_{Er}^2R_2W_0 + N_{Yb}\left(f_5^{em}R_5+R''_G\right)W_2f^{ex}\rho\sigma\right]} \quad \text{(S18)}$$

$$I_{Green} = f_5^{em}R_5N_5 = \frac{N_{Yb}^2W_2\left(f^{ex}\right)^2\rho^2\sigma^2}{N_{Er}R_2\left(1+\frac{R''_G}{f_5^{em}R_5}\right)W_0 + \frac{C_{50}N_{Er}^2R_2W_0}{f_5^{em}R_5} + N_{Yb}\left(1+\frac{R''_G}{f_5^{em}R_5}\right)W_2f^{ex}\rho\sigma} \quad \text{(S19)}$$

From Equation (18) and (19), we can see that both the I_{Red} and I_{Green} values increase with the LSPR-enhanced excitation (f^{ex}) and emission (f_4^{em} *and* f_5^{em}) fields. However, the transfer process of non-radiative energy from the excited state (R_G'' or R_R'') of $NaYF_4$:Yb-Er NP to the neighboring $W_{18}O_{49}$ NW leads to the decrease of upconversion luminescence through the competition with the LSPR-enhanced emission field ($\frac{R_R''}{f_4^{em}R_4}$ *and* $\frac{R_G''}{f_5^{em}R_5}$). Please note that this non-radiative energy transfer is very sensitive to the interaction distance between the luminescent center of $NaYF_4$:Yb-Er (Er^{3+} ion) and the plasmonic $W_{18}O_{49}$. Meanwhile, the energy transfer rate is related to the absorption intensity of $W_{18}O_{49}$ acceptor. Because the absorption intensity of $W_{18}O_{49}$ NWs in the red light region is much stronger than that in the green light region, the value of R_R'' should be much larger than that of R_G''. The finite element

method simulations indicated the similar enhancement factors on the LSPR-enhanced electric fields at green and red emissions. Thus, the I_{Red} should be much smaller than the I_{Green} for the $NaYF_4$:Yb-Er/$W_{18}O_{49}$ film due to the enhanced non-radiative process.

Moreover, from Equation (18) and (19), we can also obtain the intensity ratio of green to red emissions (I_{Green}/I_{Red}):

$$\frac{I_{Green}}{I_{Red}} = \frac{f_5^{em} R_5 N_5}{f_4^{em} R_4 N_4} = \frac{\left(1 + \frac{R_R''}{f_4^{em} R_4}\right) f_5^{em} R_5}{C_{50} N_{Er}} \tag{S20}$$

According to the results of finite element method simulations, the enhancement factor at green emission field is similar to that at the red emission field ($f_5^{em} \approx f_4^{em}$). Thus, both the non-radiative energy transfer (R_R'') and the LSPR-enhanced emission field (f_5^{em}) contribute on the enhancement of I_{Green}/I_{Red}. When the $NaYF_4$:Yb-Er NPs are in close proximity of $W_{18}O_{49}$ NWs in the $NaYF_4$:Yb-Er/$W_{18}O_{49}$ film, the values of R_R'' and f_5^{em} would increase, resulting in a very large value of I_{Green}/I_{Red}. This is one of the reasons to explain the LSPR-induced selective enhancement of upconversion luminescence on the $NaYF_4$:Yb-Er/$W_{18}O_{49}$ film.

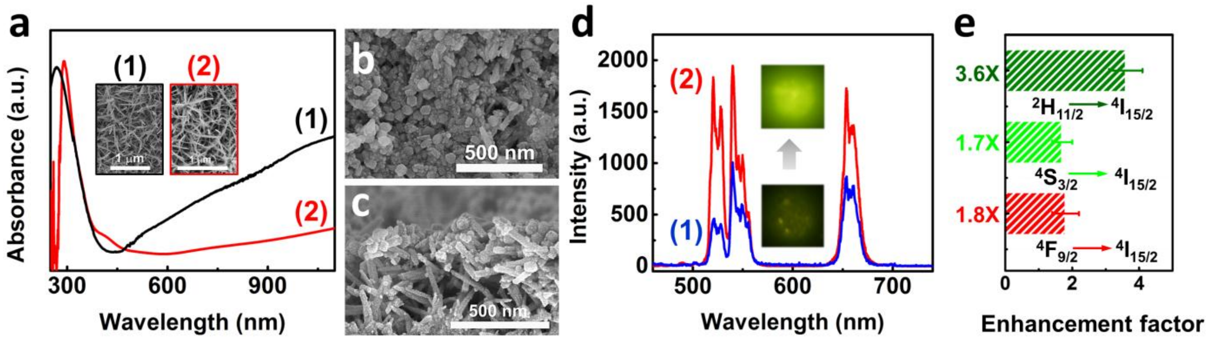

Figure S10 (a) UV-Vis-NIR absorption spectra and SEM images of the (1) $W_{18}O_{49}$ NWs and (2) W-$W_{18}O_{49}$ NWs films; SEM images of (b) the top view and (c) the side view of the $NaYF_4$:Yb-Er/W-$W_{18}O_{49}$ film; (d) upconversion emission spectra of the (1) $NaYF_4$:Yb-Er and (2) $NaYF_4$:Yb-Er/W-$W_{18}O_{49}$ films; The insets provide the corresponding micro-area optical images achieved under irradiation at 980 nm. (e) Histogram of enhancement factors calculated via the intensity ratios of upconversion luminescence of the $NaYF_4$:Yb-Er/W-$W_{18}O_{49}$ film to the $NaYF_4$:Yb-Er film at the different emission wavelengths.

To confirm the hypothesis of NIR-plasmonic energy upconversion process, we synthesized another control sample labeled as the $NaYF_4$:Yb-Er/W-$W_{18}O_{49}$ film, in which the W-$W_{18}O_{49}$ NWs only showed a weak LSPR band in NIR region. The W-$W_{18}O_{49}$ NWs were obtained by reducing the solvothermal temperature to 160℃.[5] As shown in Figure S9 a, the nanostructure of W-$W_{18}O_{49}$ NWs is the same as that of normal plasmonic $W_{18}O_{49}$ NWs on the FTO glass, while the LSPR intensity at 980 nm is remarkably lower than the intensity of normal plasmonic $W_{18}O_{49}$ NWs. Meanwhile, there is absence of LSPR in the green and red light regions for the W-$W_{18}O_{49}$ NWs, meaning that the NIR-plasmonic energy upconversion would not be realized in this $NaYF_4$:Yb-Er/W-$W_{18}O_{49}$ film. SEM images of the top surface (Figure S9 b) and cross section (Figure S9 c) of the $NaYF_4$:Yb-Er/W-$W_{18}O_{49}$ film indicated that this film had the same component morphology with the $NaYF_4$:Yb-Er/$W_{18}O_{49}$ film. Upon excitation by 980-nm, the red and green emissions of $NaYF_4$:Yb-Er/W-$W_{18}O_{49}$ film were both enhanced as compared to the pure $NaYF_4$:Yb-Er film (Figure S9 d). The enhancement factors of upconversion luminescence for $^4I_{11/2}$→$^4F_{7/2}$ (660 nm), $^4S_{3/2}$→$^4I_{15/2}$ (545 nm), and $^2I_{11/2}$→$^4I_{15/2}$ (521 nm) transitions are 1.8, 1.7, and 3.6, respectively (Figure S9 e). The enhancement factor for the $^2I_{11/2}$→$^4I_{15/2}$ (3.6×) transition is only twice higher than the factor for the $^4I_{11/2}$→$^4F_{7/2}$ (1.8×) transition. This ratio value is extremely lower than the corresponding value (90×) obtained from the $NaYF_4$:Yb-Er/$W_{18}O_{49}$ film, indicating the lack of NIR-plasmonic energy upconversion process to selectively quench the upconversion luminescence of $NaYF_4$:Yb-Er NPs. The low enhancement effect on the upconversion luminescence in this case is attributed to the weakened LSPR intensity of W-$W_{18}O_{49}$ NWs.

In the case of $NaYF_4$:Yb-Er, the energy separation of ~840 cm^{-1} can allow the $^2H_{11/2}$ level to be populated from the $^4S_{3/2}$ level by thermal excitation, and a quasi-thermal equilibrium forms between these two levels, resulting in the variation in the transitions of $^2I_{11/2}$→$^4I_{15/2}$ (521 nm) and $^4S_{3/2}$→$^4I_{15/2}$ (545 nm) at an increased temperature. The luminescence intensity ratio of the green upconversion emissions from the $^2I_{11/2}$→$^4I_{15/2}$ (521 nm) and $^4S_{3/2}$→$^4I_{15/2}$ (545 nm) transitions can be expressed as the following formula:[6]

$$R = \frac{I_H}{I_S} = \frac{I_{521}}{I_{545}} = \frac{N(\,^2H_{11/2})}{N(\,^2S_{3/2})} = \frac{g_H\sigma_H\omega_H}{g_S\sigma_S\omega_S} exp\left[\frac{-\Delta E}{kT}\right] = C\ exp\left[\frac{-\Delta E}{kT}\right] \quad \text{(S21)}$$

Where N, g, ω, and σ are the number of ions, the degeneracy, the angular frequency and the emission cross-section of luminescence transitions from the $^2I_{11/2}$ and $^4S_{3/2}$ levels to the $^4I_{15/2}$ level, respectively. ΔE is the energy separation between $^2H_{11/2}$ and $^4S_{3/2}$ levels, k is the

Boltzmann constant and T is the absolute temperature. I_H and I_S represent the integrated intensities of the transitions from the $^2I_{11/2}$ (521 nm) and $^4S_{3/2}$ (545 nm) levels to the $^4I_{15/2}$ level, respectively. It can be seen that with the increase of the local temperature surrounding the $NaYF_4$:Yb-Er NPs, the R value would increase considerably. In our case, the large ratio of the green upconversion emissions from the $^2I_{11/2}\rightarrow^4I_{15/2}$ (521 nm) and $^4S_{3/2}\rightarrow^4I_{15/2}$ (545 nm) transitions suggests the high temperature located at $NaYF_4$:Yb-Er due to the photothermal effect of $W_{18}O_{49}$ NWs. Moreover, the plasmon-induced photothermal effect of $W_{18}O_{49}$ has been also demonstrated by Lin's group (Adv. Funct. Mater. 2015, 25, 7280).

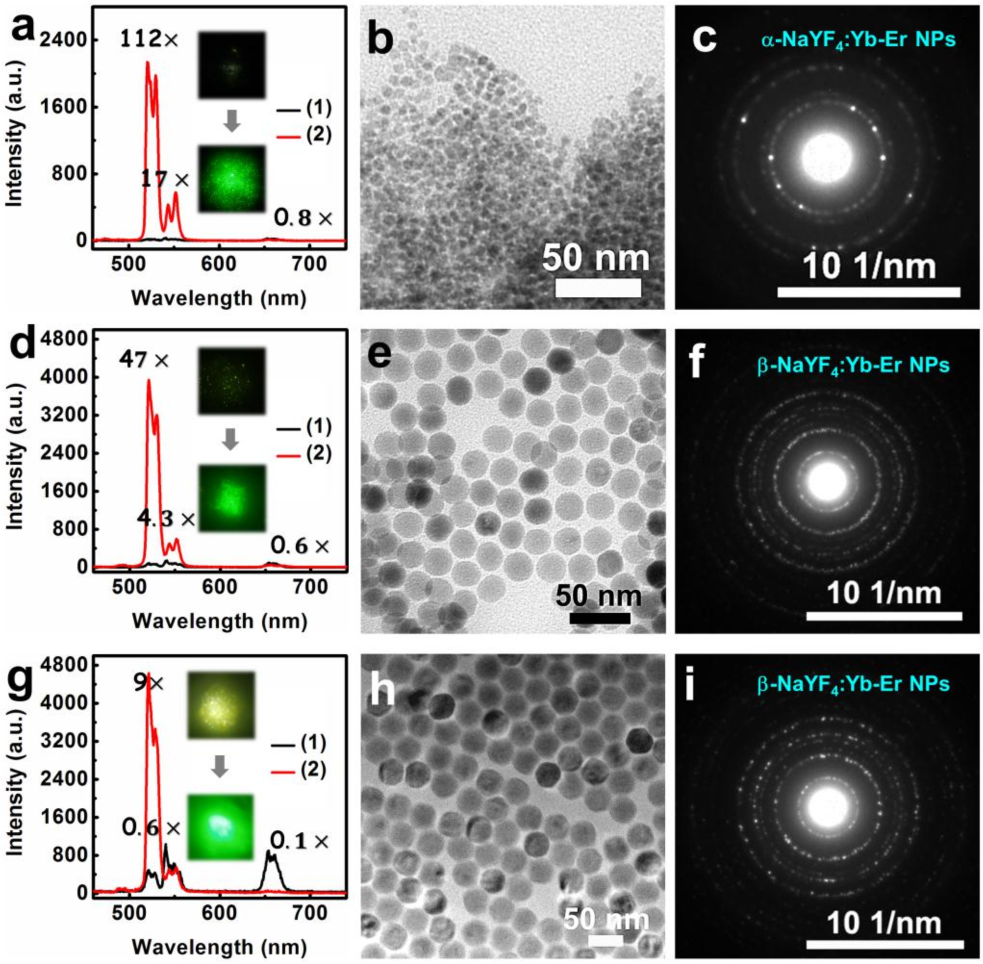

Figure S11 Upconversion emission spectra of the fabricated films loaded with different sizes of Upconversion NPs: (a) 10 nm; (d) 20 nm; (g) 40 nm: (1) $NaYF_4$:Yb-Er; (2) $NaYF_4$:Yb-Er/$W_{18}O_{49}$ film; TEM images of the Upconversion NPs with grain sizes of (b) 10 nm; (e) 20 nm; (h) 40 nm on the corresponding fabricated films; SAED patterns of the Upconversion NPs with grain sizes of (c) 10 nm; (f) 20 nm; (i) 40 nm on the corresponding fabricated films.

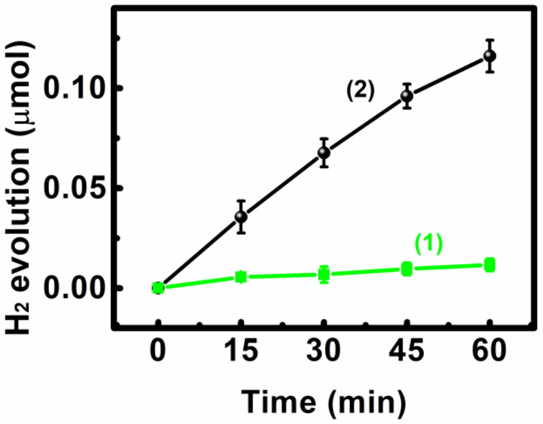

Figure S12 Plot of the H_2 evolution amount versus reaction time over (1) pure $NaYF_4$:Yb-Er upon 980-nm excitation and (2) $W_{18}O_{49}$ NWs without light irradiation in the BH_3NH_3 aqueous solution.

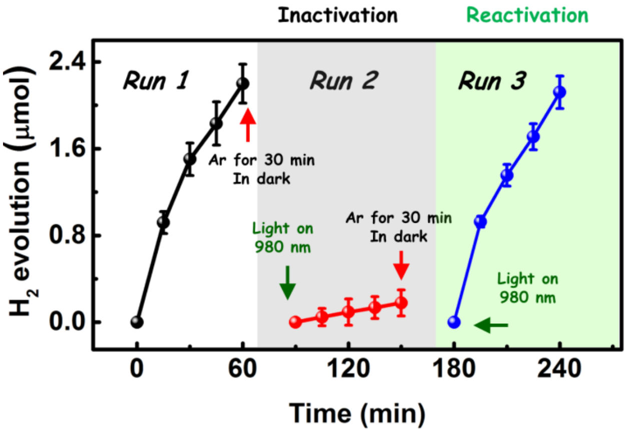

Figure S13 Cycling tests of catalytic H_2 evolution from NH_3BH_3 aqueous solution under 980-nm irradiation over the $NaYF_4$:Yb-Er@$W_{18}O_{49}$ heterostructures (Run 1 and 2) and the re-activated $NaYF_4$:Yb-Er@$W_{18}O_{49}$ heterostructures (Run 3).

The photocatalytic stability of $NaYF_4$:Yb-Er@$W_{18}O_{49}$ heterostructure was investigated under 980-nm irradiation. The result showed that after the first cycling use, the $NaYF_4$:Yb-Er@$W_{18}O_{49}$ heterostructures was almost inactive for the catalytic H_2 evolution from NH_3BH_3. The loss activity of $NaYF_4$:Yb-Er@$W_{18}O_{49}$ heterostructures can be ascribed to the quenched LSPR of $W_{18}O_{49}$ due to the reduced electron density (or surface oxygen vacancy) during the catalytic H_2 evolution. However, it has been reported that the quenched LSPR of $W_{18}O_{49}$ could be re-activated through an electrochemical treatment.[7] By using this method, we realized the reactivation of $NaYF_4$:Yb-Er@$W_{18}O_{49}$ heterostructures for H_2 evolution under 980-nm irradiation. These results also demonstrate that the LSPR-enhanced catalytic activity of $NaYF_4$:Yb-Er@$W_{18}O_{49}$ heterostructures for H_2 evolution arises mainly from the transfer of hot electron.

Furthermore, the apparent quantum efficiency (AQE) of $NaYF_4$:Yb-Er@$W_{18}O_{49}$ heterostructure was calculated through the following equation:

$$\text{AQE} = \frac{2\times number\ of\ evolved\ hydrogen\ molecules}{number\ of\ incident\ photons} \times 100\% \qquad \text{(S22)}$$

It can be calculated that the AQE of $NaYF_4$:Yb-Er@$W_{18}O_{49}$ heterostructures for H_2 evolution is ~2.8 % at 980 nm. This value is comparable (or higher) to the corresponding values obtained in other visible-light photocatalysts.[8-10]

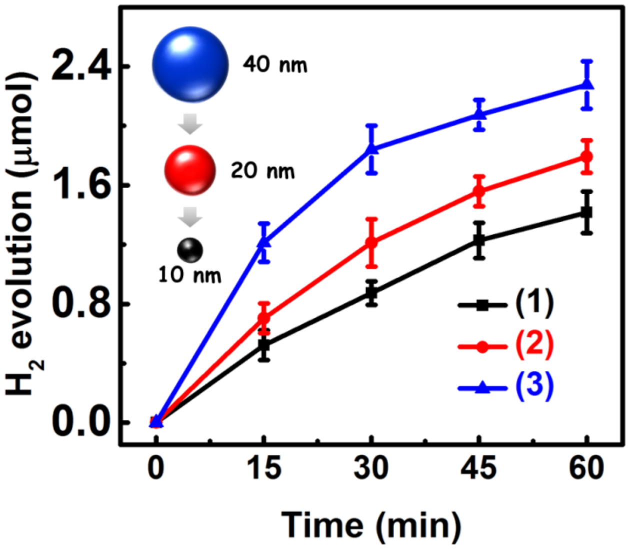

Figure S14 Time-dependent H_2 evolution from BH_3NH_3 aqueous solution upon 980-nm irradiation over $NaYF_4$:Yb-Er@$W_{18}O_{49}$ heterostructures with the sizes of $NaYF_4$:Yb-Er NPs at (1) 10 nm, (2) 20 nm, and (3) 40 nm.

References:

1 Perdew, J. P., Burke, K. & Ernzerhof, M. Generalized Gradient Approximation Made Simple. *Phys. Rev. Lett.* **77**, 3865 (1996).

2 Payne, M. C., Teter, M. P., Allan, D. C., Arias, T. A. & Joannopoulos, J. D. Iterative minimization techniques for ab initio total-energy calculations: molecular dynamics and conjugate gradients. *Rev. Mod. Phys.* **64**, 1045-1097 (1992).

3 Vanderbilt, D. Soft self-consistent pseudopotentials in a generalized eigenvalue formalism. *Phys. Rev. B* **41**, 7892 (1990).

4 Wu, D. M., García-Etxarri, A., Salleo, A. & Dionne, J. A. Plasmon-Enhanced Upconversion. *J. Phys. Chem. Lett.* **5**, 4020-4031 (2014).

5 Lou, Z., Gu, Q., Xu, L., Liao, Y. & Xue, C. Surfactant-Free Synthesis of Plasmonic Tungsten Oxide Nanowires with Visible-Light-Enhanced Hydrogen Generation from Ammonia Borane. *Chem. Asian J.* **10**, 1291-1294 (2015).

6 Dong, B. *et al.* Temperature sensing and in vivo imaging by molybdenum sensitized visible upconversion luminescence of rare-earth oxides. *Adv. Mater.* **24**, 1987-1993 (2012).

7 Zhao, J. *et al.* Trace H_2O_2 - Assisted High - Capacity Tungsten Oxide Electrochromic Batteries with Ultrafast Charging in Seconds. *Angew. Chem. Int. Ed.* **55**, 7161 (2016).

8 Gu, Q., Gao, Z. & Xue, C. Self-Sensitized Carbon Nitride Microspheres for Long-Lasting Visible-Light-Driven Hydrogen Generation. *Small* **12**, 3543-3549 (2016).

9 Yuan, Y.-P. *et al.* Improving photocatalytic hydrogen production of metal–organic framework UiO-66 octahedrons by dye-sensitization. *Applied Catalysis B: Environmental* **168-169**, 572-576, (2015).

10 Zhang, Z. *et al.* Photocatalysts: Multichannel-Improved Charge-Carrier Dynamics in Well-Designed Hetero-nanostructural Plasmonic Photocatalysts toward Highly Efficient Solar-to-Fuels Conversion. *Adv. Mater.* **27**, 5906 (2015).
